# In Vitro Cytotoxic Effect of Aqueous Extracts from Leaves and Rhizomes of the Seagrass *Posidonia oceanica* (L.) Delile on HepG2 Liver Cancer Cells: Focus on Autophagy and Apoptosis

**DOI:** 10.3390/biology12040616

**Published:** 2023-04-18

**Authors:** Giulia Abruscato, Roberto Chiarelli, Valentina Lazzara, Diletta Punginelli, Simon Sugár, Manuela Mauro, Mariangela Librizzi, Vita Di Stefano, Vincenzo Arizza, Aiti Vizzini, Mirella Vazzana, Claudio Luparello

**Affiliations:** 1 Dipartimento di Scienze e Tecnologie Biologiche Chimiche e Farmaceutiche (STEBICEF), Università di Palermo, 90128 Palermo, Italy; giulia.abruscato@unipa.it (G.A.); roberto.chiarelli@unipa.it (R.C.); valentina.lazzara@unipa.it (V.L.); diletta.punginelli@unipa.it (D.P.); manuela.mauro01@unipa.it (M.M.); librizzimariangela87@gmail.com (M.L.); vitadistefano@unipa.it (V.D.A.); vincenzo.arizza@unipa.it (V.A.); aiti.vizzini@unipa.it (A.V.); mirella.vazzana@unipa.it (M.V.); 2 MS Proteomics Research Group, Research Centre for Natural Sciences, Eötvös Loránd Research Network, 1117 Budapest, Hungary; sugarsimi@gmail.com

**Keywords:** cell biology, cell cycle, reactive oxygen species, wound healing assay, caspases, mitochondrial transmembrane potential, clonogenic assay, phenolic compounds, proteomic analysis

## Abstract

**Simple Summary:**

*Posidonia oceanica* is a widely distributed and abundant endemic seagrass in the Mediterranean area. In the attempt to identify new compounds that might inhibit specific targets in the signal transduction of liver carcinogenesis, aqueous extracts from green and brown (beached) leaves and rhizomes of the plant were prepared and tested for their cytotoxic activity on HepG2 hepatic cancer cells in culture. Here, we have identified the death-promoting mechanisms of green-leaf and rhizome extracts that involve the modulation of autophagy, apoptosis, and cell redox status, although partially differentiating conceivably due to their different compositions. These marine-derived natural materials are worth further exploration aimed at developing novel alternative prevention and/or treatment agents against liver tumors and beneficial supplements for the formulation of functional food and food-packaging material endowed with antioxidant and anticancer properties.

**Abstract:**

Aqueous extracts from *Posidonia oceanica*’s green and brown (beached) leaves and rhizomes were prepared, submitted to phenolic compound and proteomic analysis, and examined for their potential cytotoxic effect on HepG2 liver cancer cells in culture. The chosen endpoints related to survival and death were cell viability and locomotory behavior, cell-cycle analysis, apoptosis and autophagy, mitochondrial membrane polarization, and cell redox state. Here, we show that 24 h exposure to both green-leaf- and rhizome-derived extracts decreased tumor cell number in a dose–response manner, with a mean half maximal inhibitory concentration (IC_50_) estimated at 83 and 11.5 μg of dry extract/mL, respectively. Exposure to the IC_50_ of the extracts appeared to inhibit cell motility and long-term cell replicating capacity, with a more pronounced effect exerted by the rhizome-derived preparation. The underlying death-promoting mechanisms identified involved the down-regulation of autophagy, the onset of apoptosis, the decrease in the generation of reactive oxygen species, and the dissipation of mitochondrial transmembrane potential, although, at the molecular level, the two extracts appeared to elicit partially differentiating effects, conceivably due to their diverse composition. In conclusion, *P. oceanica* extracts merit further investigation to develop novel promising prevention and/or treatment agents, as well as beneficial supplements for the formulation of functional foods and food-packaging material with antioxidant and anticancer properties.

## 1. Introduction

Seas and oceans, which constitute three-quarters of the Earth’s surface, host a huge variety of organisms and represent the underexploited richest source of marine natural bioactives. Among the marine phanerogams, *Posidonia oceanica* (L.) (Delile, 1813; Liliopsida, Najadales: Posidoniacee) is the most widely distributed and abundant endemic member in the Mediterranean Sea. This slow-growing seagrass is made of buried roots and modified stems (called rhizomes, which give rise to groups of long strap-like green leaves), and forms thick and wide underwater meadows, thus colonizing vast areas accounting for about 1.5% of the surface of the Mediterranean Sea. It is listed as a protected species under various international conventions and plays a central ecological role as an indicator of the overall environmental quality of the coastal zones, including the Sicilian coastline, where it extends to 76,000 ha [1].

On the other hand, when dealing with biomedical applications, it is known that (similarly to many other aquatic species) *P. oceanica* is a reservoir full of numerous primary and secondary metabolites; therefore, extracts from this species have been the object of preclinical investigations aimed to design novel prevention and/or treatment agents effective against various pathological states. Early reports identified the antimicrobial activity of rhizome and grass extracts [2,3] and also demonstrated the dose-dependent glucose lowering and vasoprotective effects of the preparations from *P. oceanica* in a diabetic rat model [4]. In addition, the antioxidant, antiaging, antiinflammatory and antiglycation activities of the leaf extracts have been recently acknowledged [5,6,7,8]. A limited number of papers have also examined the anticancer potential of *P. oceanica* extracts. In particular, Barletta et al. [9], Leri et al. [10], and Vasarri et al. [11] reported the ability of hydrophilic preparations to inhibit gene and protein expressions of matrix metalloproteases (MMPs) in human fibrosarcoma and neuroblastoma cells through the modulation of the cells’ autophagic flux and also to block the enzymatic activity of secreted MMPs, thereby impairing cell locomotory and invasive attitudes. In addition, as shown by Farid et al. [12], different *P. oceanica* extracts exhibited varying antiproliferative activity against human breast, colon, liver, and larynx cancer cells, thus prompting the investigation of the anticancer potential of the preparations in more detail.

Hepatocellular carcinoma (HCC) is an aggressive cancer histotype that constitutes more than 90% of the cases of primary tumors of the liver and is rated as the fifth most common cancer type worldwide and the second main cause of cancer death in men [13]. HCC prognosis, which often gives rise to extensive metastasis and shows a high recurrence rate after resection or ablation, is generally poor. As reported by Philips et al. [14], in 2018, the estimated global incidence rate of liver cancer per 100,000 person-years was 9.3, while the corresponding mortality rate was 8.5. In principle, all cirrhosis-inducing conditions can increase the risk of HCC. Oxidative stress, inflammation, and continuous cycles of necrosis and regeneration are fundamental mechanisms in the development of the neoplasia and, within this altered microenvironment, the damaged hepatocytes accumulate genetic alterations and undergo progressive de-differentiation, leading to oncogenic transformation [15]. The advancement in the knowledge of the dysregulation of several signaling pathways responsible for uncontrolled cell propagation, metastasis, and recurrence of HCC cells has prompted the identification of compounds that might inhibit the specific targets in signal transduction by acting on cell cycles, apoptosis promotions, cell migratory behaviors, and other cell functions [16]. Many of these beneficial compounds are under study as potential additives for functional foods or food-packaging purposes so that their regular intake may contribute to the maintenance of the health of the organs of the digestive apparatus. However, the development of targeted therapies requires a thorough biological characterization of the molecular mechanism of action of the agents under study.

In the present study, due to the acknowledged diverse molecular composition of the different parts of *P. oceanica* seagrass [17,18], we prepared aqueous extracts from its rhizomes and leaves, both fresh (green) and beached (brown), and examined whether and to what extent each of them could exert beneficial effects on HepG2 cells in culture. This cell line, derived from the liver biopsy fragments of a 15-year-old Caucasian male affected by differentiated hepatocellular carcinoma [19], was chosen as the in vitro model system for a tumor of the digestive apparatus, being one of the most used in drug toxicity studies. The chosen biological endpoints to test the potential cytotoxic effects of the preparations on cultured cells were the viability and locomotory behavior, cell cycle distribution, apoptosis and autophagy modulation, mitochondrial membrane polarization, and cell redox state.

## 2. Materials and Methods

### 2.1. Preparation of Extracts from Green Leaves (GLE), Brown Leaves (BLE), and Rhizomes (RE) of P. oceanica 

Fresh samples of *P. oceanica* equipped with green leaves and rhizomes were collected by scuba diving off the coast of the Gulf of Palermo (Sicily, Italy), while the stranded brown leaves were collected on the coastal beach in the same place. The plant material was extensively washed under freshwater to remove the sandy residues and manually cleaned to discard the epiphytes present. For the extraction, 5 g of rhizomes (green and brown leaves) were individually powdered in a mortar in the presence of liquid nitrogen. The aqueous extracts obtained through individual resuspension of the powders in 2 M acetic acid (1:3 g/mL) plus 1:200 antiprotease cocktail (P8340, Sigma, St. Louis, MO, USA) were homogenized using a T-25 digital Ultra-Turrax instrument (IKA, Staufen, D), sonicated with an ultrasonic processor (Sonics & Materials Inc., Danbury, CT, USA), and centrifuged at 15,500 rpm for 20 min at 4 °C. The supernatants were filter-sterilized (pore size = 0.22 µm), lyophilized with an Alpha 2–4 LD plus instrument (Martin Christ, Osterode am Harz, D), resuspended in the minimum volume of sterile distilled water, and stored at −20 °C until used.

### 2.2. Chromatographic Analysis

Aliquots of 10 mg of each sample were dissolved/diluted in 1.0 mL of methanol in an autosampler vial and sonicated for 5 min. The analysis was performed with an Alliance 2695 (Waters) high pressure liquid chromatography (HPLC) system equipped with autosampler, degasser, and column heater coupled to a quadrupole time-of-flight (Waters Q-TOF Premier) mass spectrometer. The compounds were separated with a Thermo Hypersil Gold column, 5 cm, 2.1 mm, 1.9 μm particle size, at a temperature of 30 °C and with an injected volume of 5 µL. Triplicate injections of all samples were performed with a thermostatic autosampler maintained at 15 °C. For the HPLC analyses, solvent A (water, with 0.1 *v*/*v*% formic acid) was combined with solvent B (methanol, with 0.1 *v*/*v*% formic acid) under the following gradient program: from 0 to 1 min, 95% A (flow rate 0.25 mL/min); from 1 to 15 min, 100% B; from 15 to 20 min, the same percentage of solvent B; from 20 to 21 min, 100% B; from 21 to 26 min, 95% A for column re-equilibration.

The mass spectrometry (MS) experiments were performed with the dynamic range enhancement (DRE) as the acquisition mode to avoid microchannel plate (MCP) saturation and maintain a good sensitivity, thus ensuring a correct quantification of the compounds present in both high concentration and trace levels and providing more reliable results. Electrospray ionization (ESI) was utilized in a negative ion mode under the following conditions: capillary voltage, 2.0 KV; desolvation temperature, 300 °C; sampling cone, 30.0 V; extraction cone, 2.0 V; ion guide, 2 V; source temperature, 80 °C; cone gas N_2_, flow 35.0 L/h; desolvation gas N_2_, flow 300.0 L/h; scan time, 1 s; interscan delay, 0.1 s. The acquisition mass range was 100–1000 *m*/*z*; for the collection of data with an accurate mass selection; an appropriate lock mass was selected.

### 2.3. Proteomic Analysis

GLE and RE were centrifuged and the supernatants placed onto 10 kDa membrane filters for subsequent solvent exchange. Protein concentrations were determined using a NanoDrop 3300 Fluorospectrometer (Thermo Fisher, Waltham, MA, USA), then enzymatic digestion took place as previously reported elsewhere [20,21]. Briefly, the proteins in the sample were reduced using RapiGest and dithiothreitol (Thermo Fisher), then alkylated using iodoacetamide (Thermo Fisher), and finally digested using LysC-Trypsin and Trypsin (Mass Spec grade, Promega, Madison, WI, USA) in two steps. Proteolysis was stopped with the addition of formic acid (Thermo Fisher). Then, the samples were dried and desalted using C18 spin columns (Thermo Fisher) and stored at −20 °C until the analysis. Aliquots of each sample (total nominal amount = 3 µg protein) were then analyzed using an Ultimate 3000 nanoRSLC system (Dionex, Sunnyvale, CA, USA) coupled to a Bruker Maxis II ETD mass spectrometer (Bruker Daltonics GmbH, Bremen, D) via a CaptiveSpray nanobooster ion source. The peptides were separated using an ACQUITY UPLC M-Class Peptide BEH C18 column (Waters, Milford, MA, USA). Proteins were identified by searching against the Uniprot Alismatales FASTA database (accessed on 20 February 2023), using the Byonic (version 4.5.2., Protein Metrics Inc.) software search engine. Based on the results, a focused protein database was created and Scaffold was used for protein quantitation, using a 95% peptide threshold, 1% protein false discovery rate, and minimum 2 peptides per protein (version 4.11, Proteome Software). Quantitative proteomic information was provided by Scaffold’s quantitative analysis.

### 2.4. Cell Culture and Viability Assay

HepG2 liver cancer cells, taken from laboratory stocks, were grown in Dulbecco’s modified Eagle medium (D-MEM, Sigma), supplemented with 10% fetal calf serum (Sigma) and antibiotics (100 U/mL penicillin and 100 μg/mL streptomycin; Sigma) at 37 °C in a 5% CO_2_ fully humidified atmosphere. For the viability test, cells in exponential growth were seeded at a concentration of 5500/well in 96-well plates, allowed to adhere overnight, and grown in control conditions or exposed to different concentrations of either GLE, BLE, or RE ranging from 10 to 100 μg of dry extract/mL for 24 h. The number of viable cells was determined through the trypan blue exclusion test [22]. A cell viability ratio in each experimental condition was determined as the ratio between treated cells and controls; the half maximal inhibitory concentrations (IC_50_) for GLE and RE were evaluated using the CompuSyn software [23].

In parallel assays, HepG2 cells were co-exposed to both 1 nM rapamycin (Sigma) (a macrolide antibiotic that acts as an autophagy activator [24]) and IC_50_ of either GLE or RE for 24 h. Trypan blue exclusion tests were performed to monitor the reversion of the cytotoxic effect, if any. DMSO-treated cells were used as controls.

### 2.5. Clonogenic Assay

This assay was performed according to Ramos et al. [25]. Essentially, HepG2 cells were treated for 24 h with IC_50_ of GLE or RE and then detached by trypsinization and seeded in 6-well plates at a concentration of 200 cells/well with fresh unsupplemented medium. After 10 d of growth, the obtained colonies were stained with crystal violet and counted. The data from triplicate experiments were used for the determination of the plating efficiencies (PE) and the surviving fractions (SF) according to the following formulae:

PE = number of colonies counted/number of cells plated

SF = PE of exposed cells/PE of control × 100

### 2.6. Wound-Healing Assay 

This assay was performed according to Luparello et al. [20,21]. Essentially, HepG2 cells were seeded in 6-well plates at a concentration of 88,000 cells/well and, once the monolayers were confluent, the cells were scraped three times in parallel with a 200 μL pipette tip and an intersecting line was drawn with a permanent marker. The culture medium was replaced with either an unsupplemented medium (control) or IC_50_ GLE- or RE-containing media. Selected sites of intersections between the wounded cell layer and the drawn line were photographed under a phase–contrast microscope at different time points within 24 h from the start of the experiments. The wound area was quantitated using the ImageJ/Fiji^®^ plug-in and reported as mean area % ± s.e.m. of triplicate observations.

### 2.7. Flow Cytometry Assays

For each assay, cells were seeded in 6-well plates at a concentration of 88,000 cells/well and grown for the appropriate time in control conditions or exposed to IC_50_ of either GLE or RE; three independent experiments were performed as described in [20,21,26,27,28], using a FACSCanto flow cytometer (BD Biosciences, Franklin Lakes, NJ, USA) and evaluating 10,000 events. The obtained fcs files were analyzed with the online Floreada tool available at https://floreada.io (accessed on 6 March 2022). Cell debris, which displayed low FSC values, was excluded from every analysis by gating in the FSC vs. SSC plot; in addition, cell doublets and multiplets were excluded from every cell-cycle analysis by gating in the FSC-H vs. FSC-A plot.

#### 2.7.1. Cell-Cycle Analysis

The distribution of cells in the cycle phases was evaluated through fixation of control and treated cells with cold 70% ethanol, incubation with 40 μg RNase A/mL, and staining with 20 μg propidium iodide/mL.

#### 2.7.2. Apoptosis Assay

The amount of control and treated cells undergoing apoptosis was determined with the Annexin V-FITC kit (Canvax Biotech, Cordoba, Spain), following the manufacturer’s instructions. The externalization of phosphatidylserine was examined using recombinant annexin-V conjugated to green fluorescent FITC dye in conjunction with propidium iodide (PI) in order to discriminate viable, early-apoptotic, late-apoptotic, or necrotic cells

#### 2.7.3. Analysis of Mitochondrial Transmembrane Potential (MMP)

The MMP state was studied using the JC-10 Mitochondrial Membrane Potential Assay kit (Abcam, Cambridge, UK), using the cationic lipophilic JC-10 dye, which is a ratiometric fluorescent indicator sensitive to modifications of MMP. In fact, it undergoes aggregation in the mitochondrial matrix and a fluorescence emission shift from green (520 nm) to red (570 nm) in intact cells, whereas, in the case of dissipation of MMP, it remains monomeric and stains cells green. Incubation of cells with 1 μM valinomycin, a K^+^ ionophore which dissipates MMP, served as a positive control.

#### 2.7.4. Reactive Oxygen Species (ROS) Detection

The extent of intracellular ROS production in control and treated cells was determined with the ROS Detection Assay kit (Canvax Biotech), monitoring the extent of deacetylation and oxidation of the probe 2′,7′ dichlorodihydrofluorescein diacetate into the fluorescent 2′,7 dichlorodihydrofluorescein, according to the manufacturer’s instructions.

#### 2.7.5. Acidic Vesicular Organelles’ (AVO) Accumulation Analysis

The extent of AVO accumulation was evaluated through the fixation of control and treated cells with cold 70% ethanol and staining with 100 μg acridine orange/mL (Sigma) for 20 min in the dark.

### 2.8. Caspase Activity Assay

The measurement of caspase activity in cells seeded in 6-well plates at a concentration of 88,000 cells/plate and grown for 24 h in control conditions or exposed to IC_50_ of either GLE or RE was performed with the Caspase Family Colorimetric Substrate kit II Plus (Abcam), following the manufacturer’s instructions.

### 2.9. Preparation of Cell Lysates and Western Blot

Western blot analysis was performed as previously described [29]. Briefly, total protein samples were extracted from control and treated HepG2 cells (cultured to subconfluence in 25 cm^2^ flasks) in a lysis buffer (7 M Urea, 2% CHAPS, and 10 mM DTT) containing a protease inhibitor cocktail (Sigma). The protein samples (25 µg) were separated by 13% sodium dodecyl sulphate-polyacrylamide gel electrophoresis and then transferred to nitrocellulose membranes. The membranes were incubated with primary antibodies at 4 °C overnight. The antibodies used were rabbit anti-p62/SQSTM1 (P0068, Sigma; working dilution 1:500), rabbit anti-Beclin-1 (SC-11427, Santa Cruz Biotechnology, Delaware, CA, USA; working dilution 1:500), rabbit anti-LC3 (L8918, Sigma; working dilution 1:1000), mouse anti-HSP60 (H3524, Sigma; working dilution 1:500), and rabbit anti-actin (Ab8227, Abcam; working dilution 1:1000). Subsequently, the membranes were washed exhaustively and incubated with peroxidase-conjugated anti-mouse or anti-rabbit secondary antibodies (W4021 and W4011, Promega Corporation, Madison, WI, USA) at room temperature for 1 h. Protein expression was visualized using a Molecular imager Versadoc MP imaging System (Bio-Rad, Hercules, CA, USA) and the Immun Star^TM^ WesternC^TM^ chemiluminescent kit (Bio-Rad). Bands’ quantitative analysis were carried out with Quantity One v.4.6.6 software (Bio-Rad) and referred to actin band intensity, used as a loading control. The original images of the Western blots can be found as a Supplementary File (Figure S1).

### 2.10. RNA Extraction, Reverse Transcription, and Quantitative Real-Time PCR (qRT-PCR)

Total RNA samples were obtained from cells cultured to subconfluence in 25 cm^2^ flasks and grown for the appropriate time in control conditions or exposed to IC_50_ of either GLE or RE using the PureLink RNA Mini kit (ThermoFisher, Waltham, MA, USA) with on-column DNase treatment using the PureLink DNase set (ThermoFisher), following the manufacturer’s instructions. The quality and integrity of the RNAs were checked through “bleach” gel electrophoresis [30]. The cDNAs were synthesized using 10 U of Transcriptor reverse transcriptase (Roche, Manheim, Germany) and random hexamer primers according to the manufacturer’s instructions; their quality was checked through PCR amplification in the presence of 1 U/μL of Taq DNA polymerase (TAQ-RO, Roche) and the forward and reverse primers for β-actin, following the manufacturer’s instructions. 

Differential expression of *BAD*, *BAX*, *BCL2*, *death-associated protein kinase (DAPK)*, *JUN,* and *FOS* genes was tested through qRT-PCR using the SYBR-Green method, the specific sets of primers listed in Table 1, and an Applied Biosystems 7500 Real-Time PCR system. The 25 µL PCR mixtures contained 2 µL of cDNA reverse transcribed from 250 ng of total RNA, 300 nM primers (forward and reverse), and 12.5 μL of Power SYBR-Green PCR MasterMix (Applied Biosystems, Waltham, MA, USA). The specificity of amplification was tested by real-time PCR melting analysis. To obtain sample quantification, the 2^−ΔΔCt^ method was used, as described in the Applied Biosystems Use Bulletin N.2 (P/N 4303859); the transcript levels were normalized to that of β-actin to compensate for variations in the amount of RNA input. The relative expression was evaluated as the ratio between the normalized value of the target gene in each treated sample and the normalized value obtained from the samples in control conditions. 

### 2.11. Statistics

The normality tests were performed with SigmaPlot 11.0 software (SYSTAT, San Jose, CA, USA). For Western blot experiments, data were analyzed by unpaired two-tailed Student’s t-test with GraphPad Prism 9 software (GraphPad, San Diego, CA, USA).

## 3. Results

### 3.1. Characterization of GLE, BLE, and RE

Polyphenols were evaluated in GLE, BLE, and RE; the results are shown in Table 2. Not all polyphenols identified were quantifiable, probably due to the extraction method used; these compounds were indicated as n.q. Interestingly, in several cases, the three preparations showed a different content of polyphenols. Higher levels of polyphenols were observed in RE and, in detail, we observed a conspicuous amount of delphinidin-3-glucoside and quercetin 3-O-galactoside and a much lesser, but still measurable, concentration of vanillic acid and procyanidins B2 and -3. In GLE and BLE, on the other hand, the most represented polyphenols were caffeic acid methyl ester and p-hydroxybenzoic acid, respectively.

### 3.2. Short-Term Viability and Long-Term Proliferative Potential of HepG2 Cells Exposed to P. oceanica Extracts

First, the cytotoxic effects of GLE, BLE, and RE were examined via trypan blue exclusion assay and, in case of dose-dependent viability inhibition, the specific IC_50_ was determined. Cell exposure for 24 h to GLE and RE resulted in a concentration-dependent decrease in cell viability, whereas treatment with BLE at all the concentrations tested was ineffective at modifying the percentage of viable cells and therefore the latter preparation was excluded from the subsequent analyses. The mean IC_50_ was estimated as 83 and 11.5 μg of dry extract/mL for GLE and RE, respectively; such concentrations were chosen for all the following experiments, aimed at deepening the knowledge on some aspects of extract-induced cytotoxicity.

**Figure 1:**
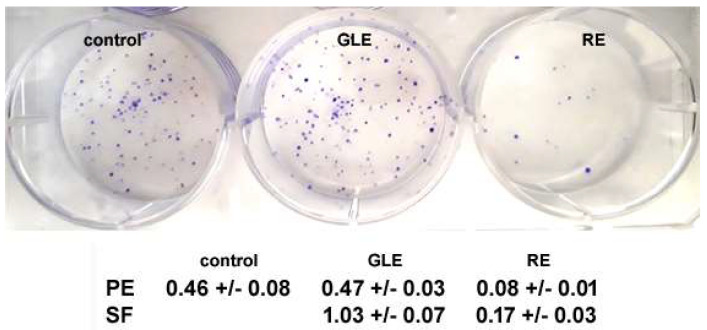
Representative images of clonogenic assays of HepG2 cells in control conditions or exposed to the IC_50_ of GLE or RE for 10 days. The corresponding plating efficiencies (PE) and surviving fractions (SF) are reported in the annexed table (mean ± s.e.m. of three independent experiments).

In a second set of assays, the capacity for continued proliferation or, in contrast, the onset of cell reproductive death after the treatments with GLE or RE, were investigated by submitting HepG2 cells to clonogenic assay (also known as colony formation assay), aimed at evaluating the long-term ability of single cells to grow into a colony of at least 50 individuals after 24 h exposure to the IC50 of the extracts [35]. Representative micrographs of the stained colonies and the PE and SF values in either experimental condition, obtained from colony count in triplicate experiments, are shown in Figure 1.

The results indicate that, differently from cell exposure to GLE that was ineffective in modifying cell behavior, the exposure to RE determined a significant reduction of cells’ replicating capacity, down to about 16% of that of the untreated control, thereby suggesting the occurrence of phenotypic effects that are conceivably spread over time and develop after a number of cell divisions.

### 3.3. Cell-Cycle Status and Apoptosis Induction in HepG2 Cells Exposed to P. oceanica Extracts

**Figure 2:**
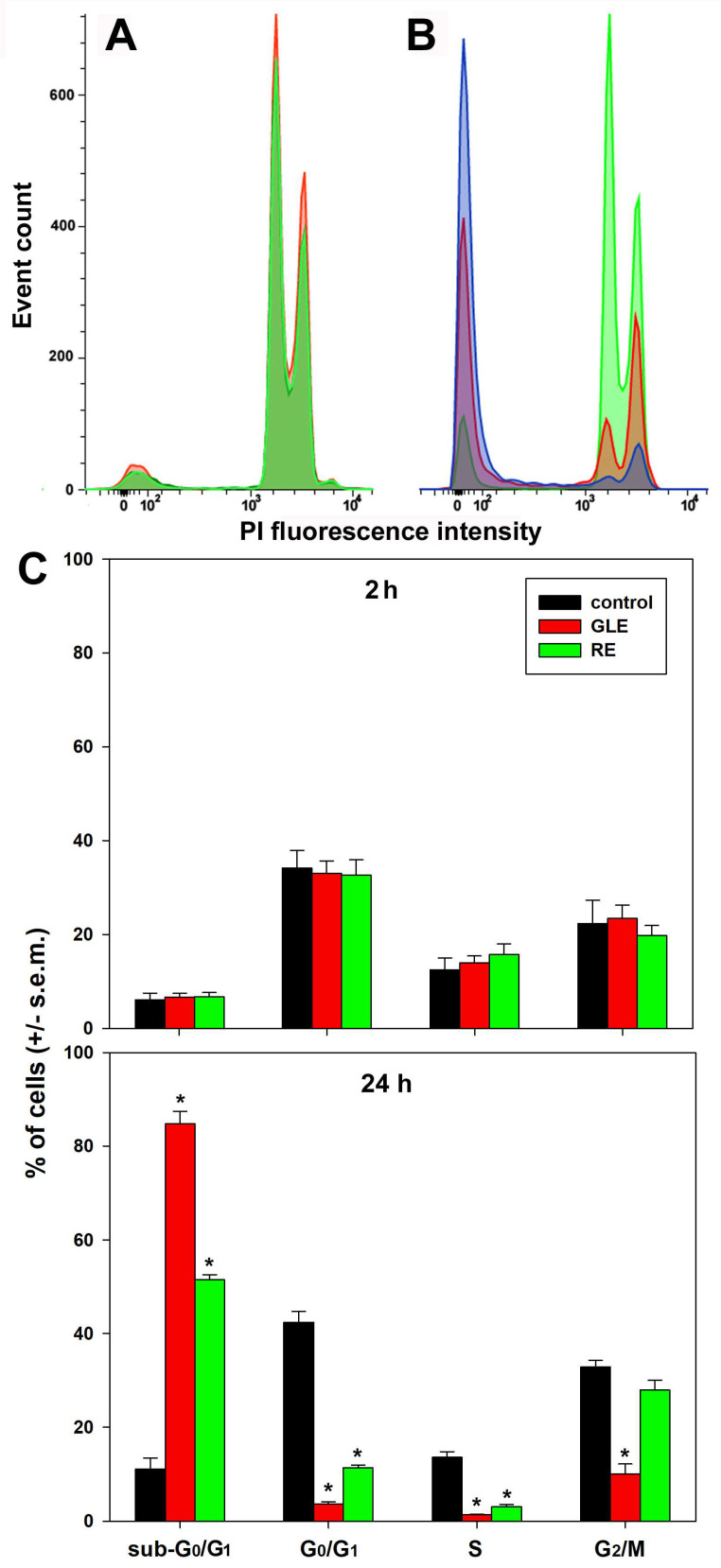
Representative DNA profiles of control (green), GLE (blue), and RE-treated (red) HepG2 cells after 2 (**A**) and 24 h (**B**) of exposure. (**C**) Bar graphs showing the effect of GLE and RE on the cell-cycle distribution of HepG2 cells after 2 and 24 h of treatment compared with controls. The error bars indicate the standard error of the mean (s.e.m.) of three independent measurements. * normality test vs. control passed.

In the light of the observed reduction of cell viability upon short-term treatment with the extracts, we evaluated the distribution of HepG2 cells along the phases of the cell cycle in control conditions and after 2 and 24 h of exposure to either GLE or RE. As shown in Figure 2, the 2 h treatments of cells did not produce any significant variation of the percentages of the cell population in the cycle phases. Conversely, after 24 h of exposure, we detected a shared accumulation of cells at the sub-G_0_/G_1_ stage (more pronounced in the case of exposure to GLE) and a decrease in the proportion of cells at the other stages, except for GLE-treated cells at the G_2_/M stage, whose amount remained significant and comparable to that of the controls. This suggests that, differently from the generalized RE-induced cell damage spanning the whole cycle, GLE treatment might determine a sustained impairment at the G_2_ DNA checkpoint, with a consequent arrest of the cycle progress at G_2_/M, which is an event commonly preceding apoptosis of HepG2 [36,37].

**Figure 3:**
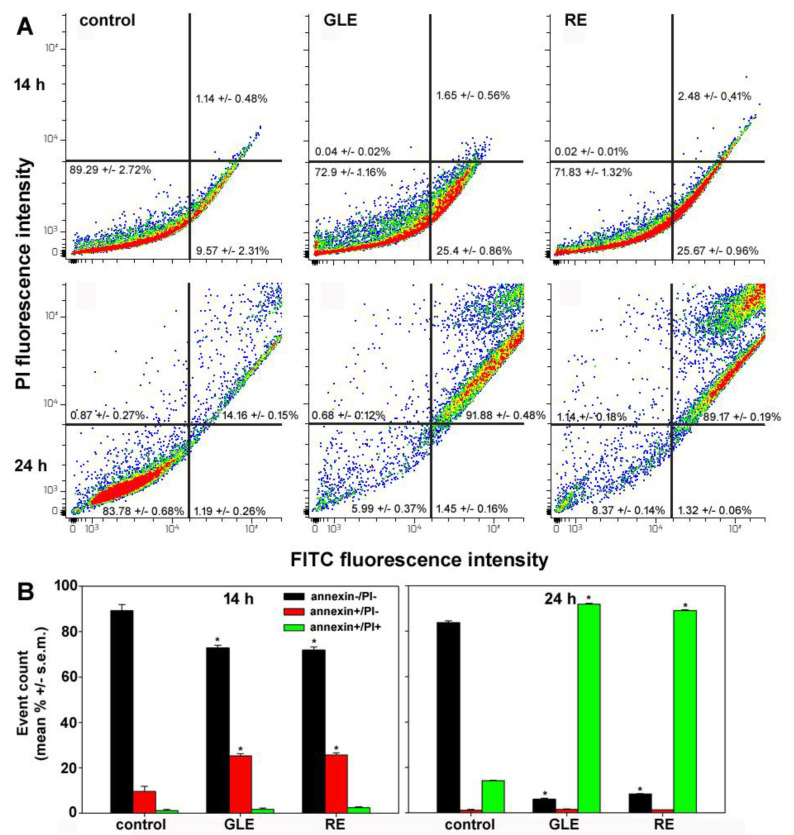
(**A**) Flow cytometric assays for apoptosis in HepG2 cells cultured in control conditions or exposed to the IC_50_ of GLE or RE for 14 or 24 h. The plots show the results of representative experiments and the percentages, indicated as the mean ± s.e.m. of three independent experiments, refer to viable annexin-V^−^/PI^−^ cells (bottom left quadrant), early apoptotic annexin-V^+^/PI^−^ cells (bottom right quadrant), late apoptotic annexin-V^+^/PI^+^ cells (top right quadrant), and necrotic annexin-V^−^/PI^+^ cells (top left quadrant). (**B**) Bar graphs summarizing the data for live, early apoptotic and late apoptotic control and treated cells obtained from triplicate experiments. * normality test vs. control passed.

Then, we examined whether the observed derangement of cell-cycle progress and increase in the proportion of sub-G_0_/G_1_ hypodiploid cells could be, at least in part, ascribed to an apoptosis-promoting effect of the extracts on HepG2 cells. Cultures grown in control conditions or exposed to GLE or RE for 14 or 24 h were assayed for the externalization of phosphatidylserine in conjunction with PI staining; the obtained data are shown in Figure 3. The data obtained indicated that, at an early stage of exposure (14 h), the percentage of the viable annexin-V^-^/PI^-^ cells decreased from about 89% of the controls to about 72% after both treatments. On the other hand, the percentage of early apoptotic cells (annexin-V^+^/PI^−^) increased from about 9% of the controls to about 25%. As expected, after 24 h of exposure, the number of viable cells was drastically reduced by about 6 (for GLE) and 8% (for RE), whereas the proportion of late apoptotic cells (annexin-V^+^/PI^+^) increased to about 92 (for GLE) and 89% (for RE) of the population. This result is consistent with the flow cytometric data regarding the observed accumulation of cells in the sub-G_0_/G_1_ fraction after 24 h of exposure to the extracts. No significant difference was found among the necrotic, i.e., annexin-V^−^/PI^+^, cell populations in the experimental conditions under study.

**Figure 4:**
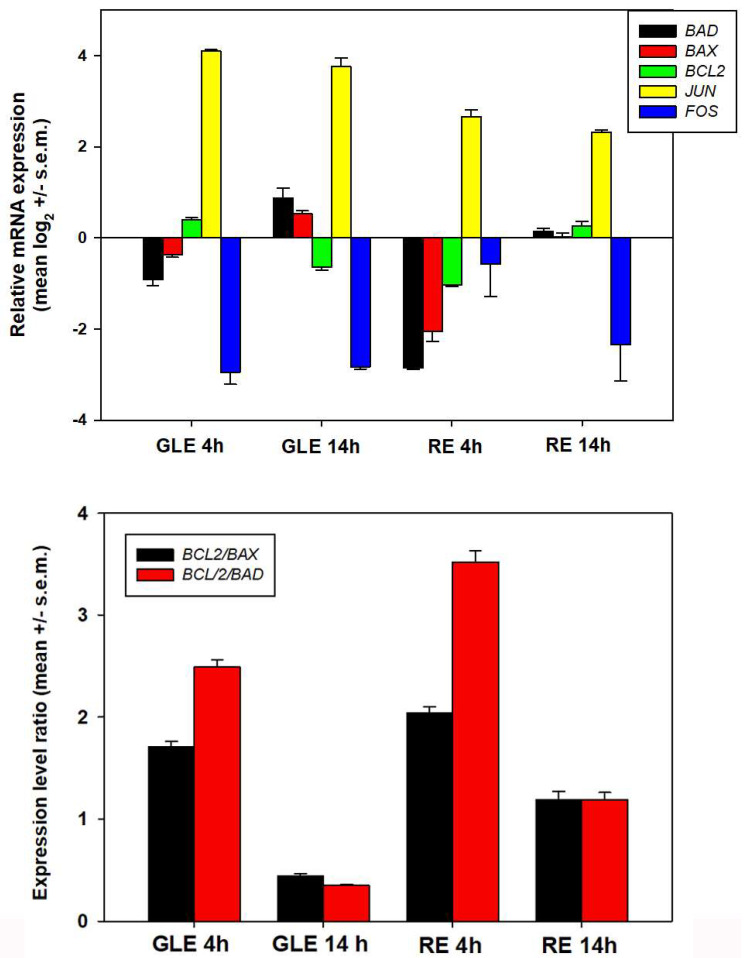
(Upper graph) real-time PCR analysis of BAD, BAX, BCL2, JUN, and FOS expression in HepG2 cells cultured in control conditions or exposed to the IC_50_ of GLE or RE for 4 and 14 h. Gene expression values are normalized relative to ACTB. (Lower graph) BCL2/BAX and BCL2/BAD expression ratios in HepG2 cells cultured in control conditions or exposed to the IC_50_ of GLE or RE for 4 and 14 h. The error bars indicate the standard error of the mean (s.e.m.) of three independent measurements. All the data passed the normality test vs. control.

Real-time PCR analyses were performed to detect the changes of Bcl-2, Bax, Bad, Fos, and Jun mRNA transcription at 4 and 14 h of exposure to GLE and RE (Figure 4).

As suggested by Golestani Eimani et al. [38], since the balance between anti- (Bcl-2) and pro-apoptotic members (Bax and Bad) of the Bcl-2 family [39] is a much better indicator of the sensitivity to apoptosis, we focused on the Bcl-2/Bax and Bcl-2/Bad expression ratios instead of the individual expression patterns. Under the experimental conditions used, the >1 ratios found with both extracts after 4 h of treatment were lowered after 14 h of culture, more drastically in the presence of GLE. In particular, the Bcl-2/Bax ratio decreased from 1.71 to 0.44 with GLE and from 2.04 to 1.19 with RE, whereas the Bcl-2/Bad ratio decreased from 2.49 to 0.35 with GLE and from 3.52 to 1.19 with RE. It is generally acknowledged that the products of the immediate early genes FOS and JUN, which are involved in the formation of AP-1 transcription factor, control various cell functions, including apoptosis [40]. In particular, the decrease in the FOS expression level was found to be associated with cell death induced by the suppression of expression of Cyclin D1 and G_0_/G_1_ arrest in colorectal cancer cells and also with c-Jun/ATF2-mediated neuronal apoptosis, whereas JUN up-regulation was responsible for caspase activation and apoptosis induction in myeloma cells [41,42,43]. Dealing with HepG2 cells, JUN overexpression was proven to down-regulate cyclin A2, thus leading to G_1_ arrest [44]. In liver tissue, a decline in FOS expression with sustained high levels of JUN expression signaled for irreversible damage and correlated with the onset of programmed hepatocellular death [45]. As shown in the figure, and in line with the mentioned reports, the up-regulation of JUN and concurrent down-regulation of FOS were detected at early and late times of exposure after treatment with both extracts. 

**Figure 5:**
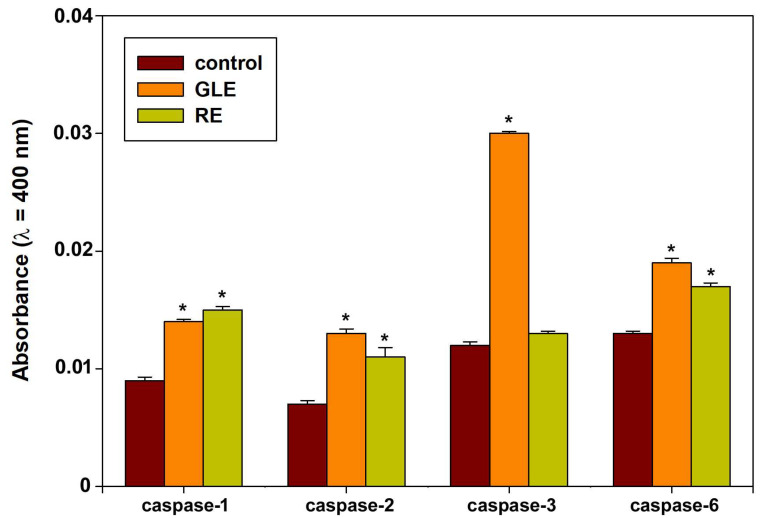
Caspase activity determination in HepG2 cells cultured in control conditions or exposed to the IC_50_ of GLE or RE for 24 h. The error bars indicate the standard error of the mean (s.e.m.) of three independent measurements. * normality test vs. control passed.

Further, to obtain more details about the activation of specific caspases following exposures, we submitted control and treated cell lysates to spectrophotometric measurements in the presence of p-NA conjugated substrates for caspase-1 to -6 and -8 to -9. Figure 5 shows that exposure to both extracts determined the activation of caspase-1, -2, and -6, whereas, in the presence of the sole GLE, the additional and prominent activation of caspase-3 was observed. No statistically relevant result was obtained for caspase-4, -5, -8, or -9.

### 3.4. Mitochondrial Membrane Polarization and Redox Status in HepG2 Cells Exposed to P. oceanica Extracts

**Figure 6:**
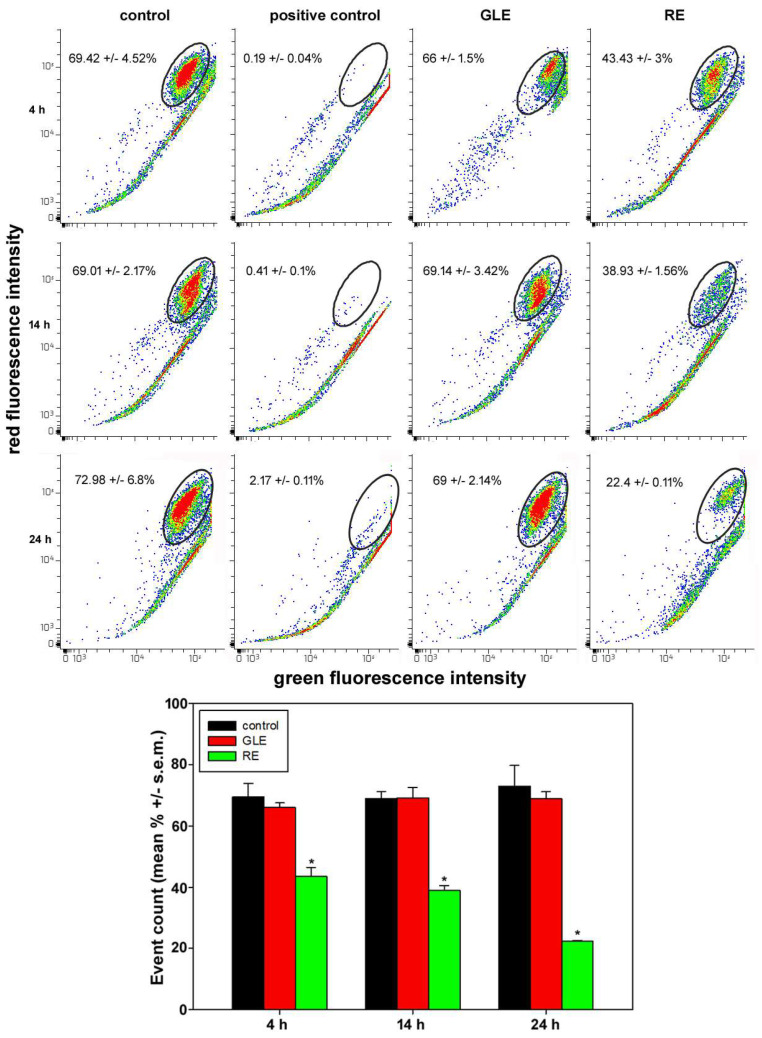
(Upper panel) flow cytometric assays for MMP in HepG2 cells cultured for 4, 14, and 24 in control conditions or exposed to 1 μM valinomycin (positive control) or the IC_50_ of either GLE or RE. The plots show the results of representative experiments, and the percentages in each frame, indicated as the mean ± s.e.m. of three independent assays, are referred to the intense red-emitting cells with intact MMP in the gated area. (Lower image) bar graph summarizing the data for control and treated cells with intact MMP obtained from triplicate experiments. * normality test vs. control passed.

We explored whether cell exposure to either GLE or RE could affect the mitochondrial function by examining the MMP status with the cationic lipophilic JC10 dye. Thus, we evaluated the percentage of cells with either intense red or bright green/dim red fluorescence (indicative of intact or collapsed MMP, respectively) after 4, 14, and 24 h from the start of the assay. Figure 6 shows that, differently from GLE, treatment with RE determined the gradual increase in the population of cells, exhibiting mitochondrial depolarization. In particular, in the three time intervals considered, the dim red-emitting cells increased from about 31, 31, and 28% of the controls to about 57, 61, and 78%, respectively. However, in no experimental condition did the values obtained increase up to those of the valinomycin-treated positive controls (98–99%), indicating the persistence of a HepG2 cell subpopulation endowed with intact mitochondrial functions.

**Figure 7:**
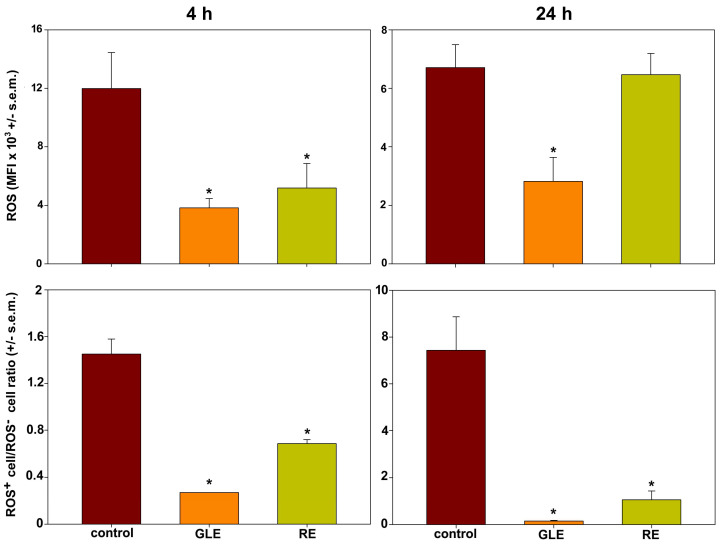
Histograms showing the ROS-associated MFI in ROS^+^ subpopulations (upper graphs) and the ROS^+^ cell/ROS^-^ cell ratio (lower graphs) of HepG2 cells grown in control conditions or exposed to the IC_50_ of GLE or RE for 4 and 24 h. The error bars indicate the standard error of the mean (s.e.m.) of three independent measurements. * normality test vs. control passed.

It is known that mitochondria are the primary source of intracellular ROS, although these can be produced also by other cellular components, such as endoplasmic reticulum-bound enzymes [46,47]. On the other hand, HepG2 cells represent a useful model to examine the production of ROS mainly from mitochondrial sources, since cytochrome P450 family 2 subfamily E member 1 (CYP2E1), a ROS-generating enzyme of the endoplasmic reticulum, is poorly expressed [48]. The accumulation of diverse types of ROS (hydrogen peroxide, peroxynitrite, hydroxyl radicals, nitric oxide, and peroxy radicals) after 4 and 24 h of exposure to either GLE or RE was examined by flow cytometry. As reported for other experimental models [20,21,49,50], in each condition studied, two distinct cell subpopulations endowed with low (ROS^−^) and high rates (ROS^+^) of ROS generation were found. The mean fluorescence intensity (MFI) values of the events associated with the ROS^+^ subpopulations, and also the overall proportion of ROS^+^ cells within the entire populations, were calculated and reported in Figure 7. A down-regulation in ROS generation could be observed already after 4 h of incubation with both extracts with an average 69% (for GLE) and 57% (for RE) decrease in exposed cells vs. controls, respectively (average control cells’ MFI = 11,989, average GLE-treated cells’ MFI = 3831, average RE-treated cells’ MFI = 5184; *p* < 0.05). The reduction in ROS quantity appeared to be reverted after 24 h of treatment with the sole RE (average control cells’ MFI = 6710, average treated cells’ MFI = 6465; no statistical significance), whereas the level of ROS produced in GLE-treated HepG2 cells remained lower than that of control cells, although to a slightly lesser degree, i.e., 58% (average control cells’ MFI = 6710, average treated cells’ MFI = 2821; *p* < 0.05). Moreover, the proportions of the ROS^+^ subpopulations of treated cells were always smaller than those of controls and showed a decline over the time interval studied, accounting for about 19% (at 4 h) and 2% (at 24 h) with GLE and about 47% (at 4 h) and 14% (at 24 h) with RE, as expected from the progressive advancement of cell impairment and death.

### 3.5. Indices of Autophagic Activity in HepG2 Cells Exposed to P. oceanica Extracts

We next examined whether the extracts could affect the autophagic flux of HepG2 cells by analyzing in parallel the accumulation of AVOs and the intracellular abundance of the protein markers LC3, Beclin-1, p62/SQSTM1, and hsp60 at early (4 h), intermediate (14 h), and late (24 h) times of exposure. LC3 protein is known to be anchored in the autophagosome membrane after the conversion of LC3-I (cytoplasmic form) to LC3-II (autophagosome membrane-associated form). Beclin-1 is known to regulate autophagy, being an essential effector that plays important roles in the crosstalk with the apoptosis pathway. The dissociation of Beclin-1 from Bcl-2 is essential for its autophagic activity. In contrast to LC3, which is a marker of final autophagosome formation, Beclin-1 participates in the early stages of autophagy, promoting the nucleation of the autophagic vesicle and recruiting proteins from the cytosol. P62/SQSTM1 protein is commonly found in the inclusion bodies containing polyubiquitinated protein and provides important information about the proteins destined for autophagic degradation. Finally, considering the involvement of polyubiquitinated proteins destined for autophagic degradation, as indicated by p62/SQSTM1, we monitored the amount of hsp60, an indicator of cytoprotection, being a component of the machinery for the correct folding of proteins [51,52,53,54]. Additionally, the expression levels of the DAPK gene were monitored at early and intermediate times of exposure by real-time PCR. It is known, in fact, that DAPK expression in various cell lines, including HepG2, leads to the enhanced formation, trafficking, and fusion of autophagosomes and also to the increase of Beclin-1 levels, thereby being considered a powerful and unique inducer of the autophagic flux [55,56].

**Figure 8:**
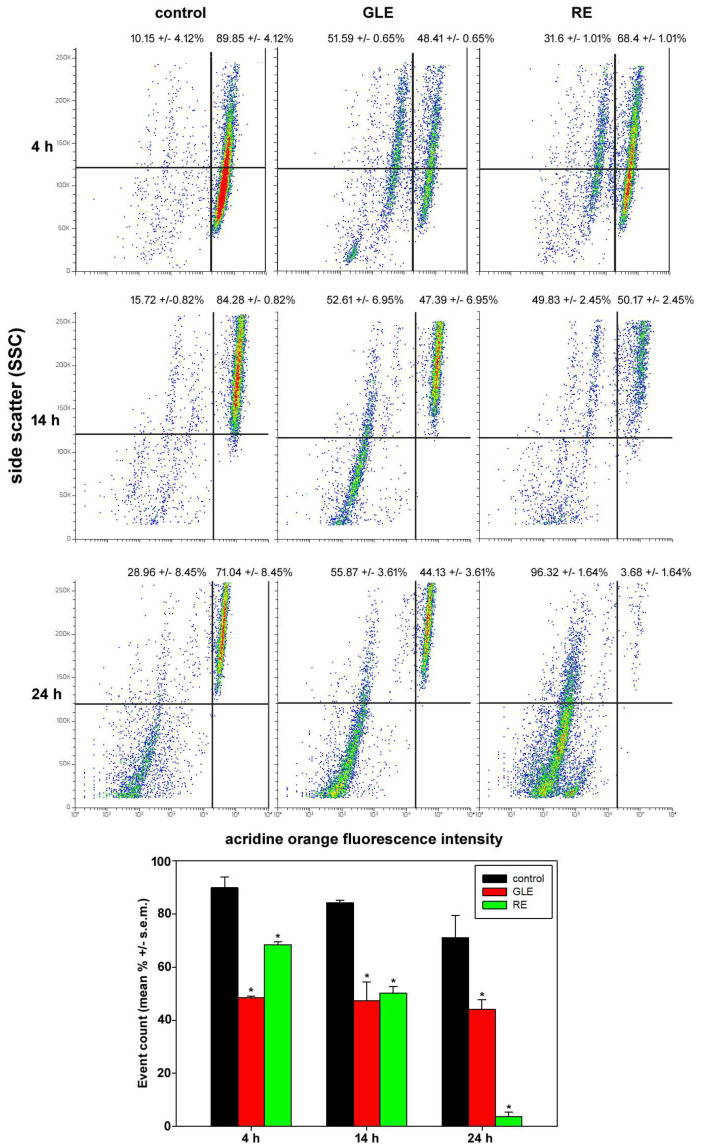
(Upper panel) flow cytometric assays for AVOs in HepG2 cells cultured in control conditions or exposed to the IC_50_ of GLE or RE for 4, 14, and 24 h. The plots show the results of representative experiments and the percentages, indicated as the mean ± s.e.m. of three independent experiments, refer to AVO^+^ cells (right quadrants) and AVO^-^ cells (left quadrants). (Lower image) bar graph summarizing the data for control and treated AVO^+^ cells obtained from triplicate experiments. * normality test vs. control passed.

As shown in Figure 8, two distinct cell subpopulations displaying low (AVO^-^) and high rate (AVO^+^) of acridine orange fluorescence were observed in the plot analyses. It is acknowledged that HepG2 cells are endowed with an elevated basal level of autophagy [20,21] and, accordingly, the percentage of AVO^+^ cells accounted for about 90, 84, and 71% at 4, 14, and 24 h of culture in untreated conditions. Following exposure to IC_50_24 GLE or RE, two different results were obtained. In fact, in the first experimental condition, the proportion of AVO^+^ cells after 4 h decreased to about 48% and the extension of the time of exposure to 14 and 24 h did not cause any further significant decrease in this percentage (about 47% after 14 h; about 44% after 24 h); conversely, exposure to RE determined the time-dependent almost complete disappearance of the AVO^+^ cell fraction, whose amount dropped to about 68, 50, and 4% of the total population after 4, 14, and 24 h of treatment.

**Figure 9:**
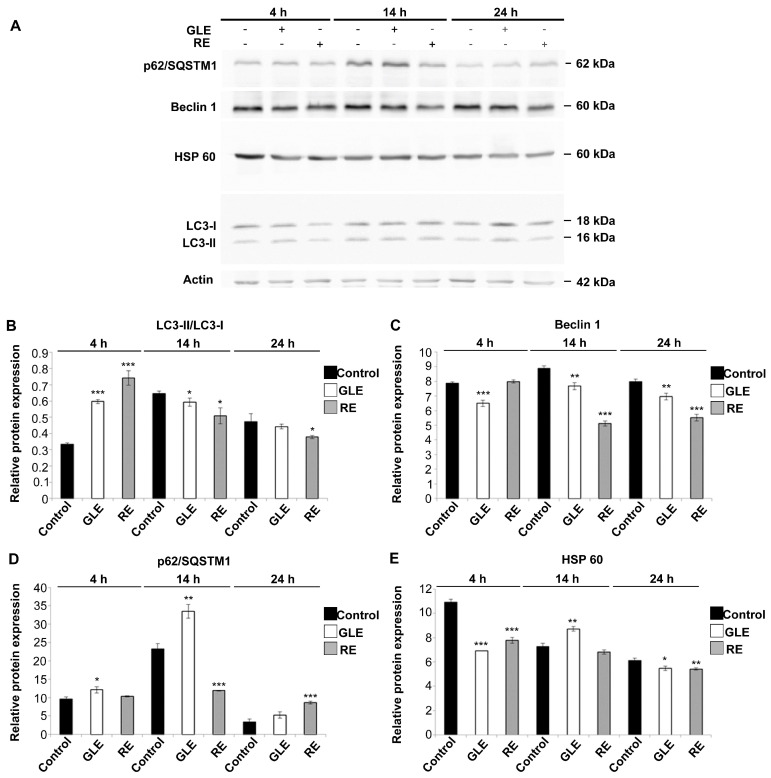
Immunoblotting detection and quantitative analysis. (**A**) Total lysates of control and GLE- or RE- treated cells after 4 h, 14 h, and 24 h of exposure, immunoreacted with anti-p62/SQSTM1, -Beclin-1, -HSP 60, -LC3, and -actin antibodies. The histograms show the densitometric analysis of the obtained bands for (**B**) LC3-II/LC3-I ratio, (**C**) Beclin-1, (**D**) p62/SQSTM1, and (**E**) HSP 60. Actin was used as the loading control and, in C-E, the relative protein expression, reported as arbitrary units, was calculated as the band density ratio to that of actin. The experiments were performed in triplicate and the data are expressed as means ± standard deviation (n = 3 ± SD). * *p* ≤ 0.05; ** *p* ≤ 0.01; *** *p* ≤ 0.005 vs. control for each time of treatment.

The Western blot data on the expression levels of autophagy- and cytoprotection-related proteins are shown in Figure 9. We analyzed the LC3-II/LC3-I ratio in order to monitor the level of the autophagic flux during the relative times of exposure. The results indicate that, after 4 h, an initial up-regulation of the ratio was observed after both exposures, being approximately 1.8- and 2.2-fold higher for GLE- and RE-treated cells, respectively, compared with controls, followed by an approximate 0.1- and 0.2-fold decrease at 14 h from the start of the assay. After 24 h, a further slight decline of the ratios was observed under both experimental conditions (GLE-treated cells = about 0.1-fold; RE-treated cells = about 0.2-fold). Concerning Beclin-1, after 4 h of treatment with GLE, its level appeared to be about 0.8-fold lower than that of the control; a comparable level of Beclin-1 was instead found in the presence of RE. After 14 h, on the other hand, Beclin-1 was down-regulated in both treated samples, although to different degrees and in a more pronounced manner in cells treated with RE (about 0.4-fold). The same trend was also observed after 24 h under both experimental conditions. After 4 h of treatment, the level of p62/SQSTM1 appeared to increase by about 0.3-fold for GLE-treated cells compared with the untreated sample, while in RE-treated cells it was comparable to that of the control. Similarly, after 14 h, time during which physiologically the control cells expressed high levels of p62/SQSTM1, the increase in this protein was higher for GLE- (about 0.4-fold) and lower for RE-treated cells (about 0.5-fold) with respect to control cells. This treatment period concurred, however, with the highest levels of p62/SQSTM1 in the whole experiment. After 24 h, low levels of p62/SQSTM1 were found in the controls compared with the treated samples, showing a larger increase in RE- (about 1.5-fold) than GLE-treated cells (about 0.5-fold). Overall, it is of note that p62/SQSTM1 levels in RE-treated cells remained constantly low in the three times of exposure. Finally, concerning hsp60, its levels were reduced after 4 h of treatment compared with the control, more prominently in GLE-treated cells (about 0.4-fold). After 14 h, the level of hsp60 was elevated by about 0.2-fold with respect to the control only in the presence of GLE, whereas it remained comparable to the control for RE-treated cells. Finally, after 24 h, hsp60 was reduced by about 0.9-fold in both treated conditions. 

**Figure 10:**
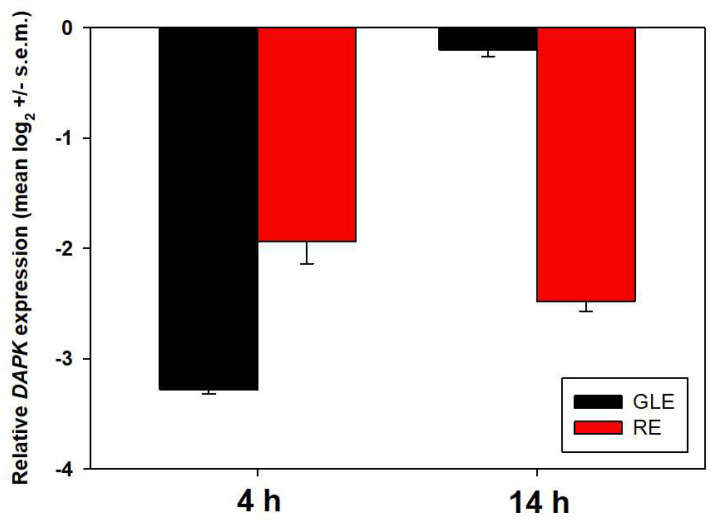
Real-time PCR analysis of DAPK expression in HepG2 cells cultured in control conditions or exposed to the IC_50_ of GLE or RE for 4 and 14 h. Gene expression values are normalized relative to ACTB. The error bars indicate the standard error of the mean (s.e.m.) of three independent measurements. All the data passed the normality test vs. control.

As expected from the different extents of autophagy down-regulation indicated by the flow cytometric and protein blot data, different patterns of DAPK expression were found after treatments with either preparation, confirming the previous results. In particular, a drastic decrease in its transcription rate was observed at 4 h of exposure in the presence of both GLE and RE. Consistent with the gradual total disappearance of the AVO^+^ cell population, the prominent down-regulation of DAPK remained stable within 14 h of treatment with RE, whereas, in line with the stabilization of AVO^+^ cells to values equal to about 50% of the total population, at 14 h of exposure to GLE, the amount of DAPK mRNA returned to values slightly lower than those of control cells (Figure 10). Lastly, the increase in c-Jun transcription, which has been reported in our experimental system, is considered as a prelude to autophagy inhibition [57]. 

**Figure 11:**
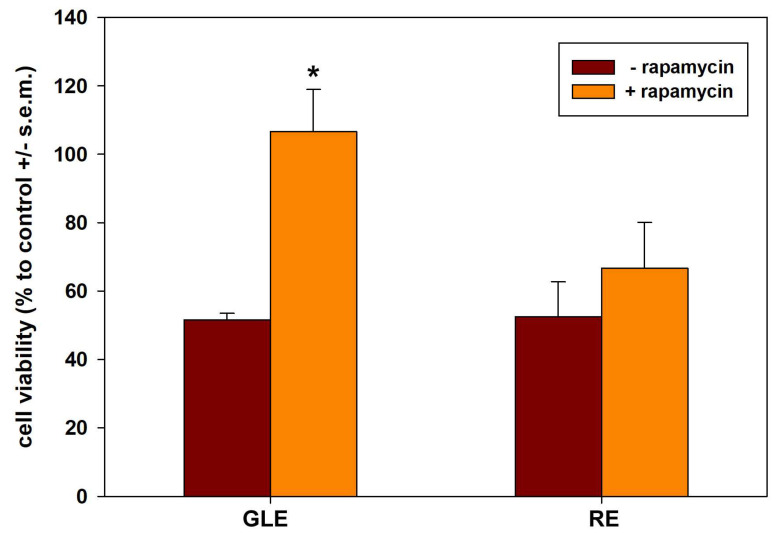
Histograms showing the effect exerted by 1 nM rapamycin co-treatment on GLE- or RE-induced decrease in HepG2 cell viability after 24 h of exposure. The error bars correspond to the standard error of the mean (s.e.m.) of three independent measurements. * normality test vs. control passed.

Then, we examined to what extent the inhibition of the autophagic process could be responsible for the observed GLE- and RE-dependent cytotoxic effect on HepG2 cells. To this purpose, cells were co-incubated with IC_50_24 of either GLE or RE and 1 nM of the autophagy-stimulator rapamycin (sirolimus), which acts through the inhibition of mTOR (mammalian target of rapamycin) serine/threonine protein kinase. As shown in Figure 11, the co-exposure was capable of significantly reversing the decrease in cell number only in the presence of GLE; conversely, no significant effect was found after RE-rapamycin co-administration, thereby confirming the occurrence of more extensive and widespread damage induced by exposure of HepG2 cells to this specific preparation.

### 3.6. Inhibition of the Locomotory Ability of HepG2 Cells Exposed to P. oceanica Extracts

**Figure 12:**
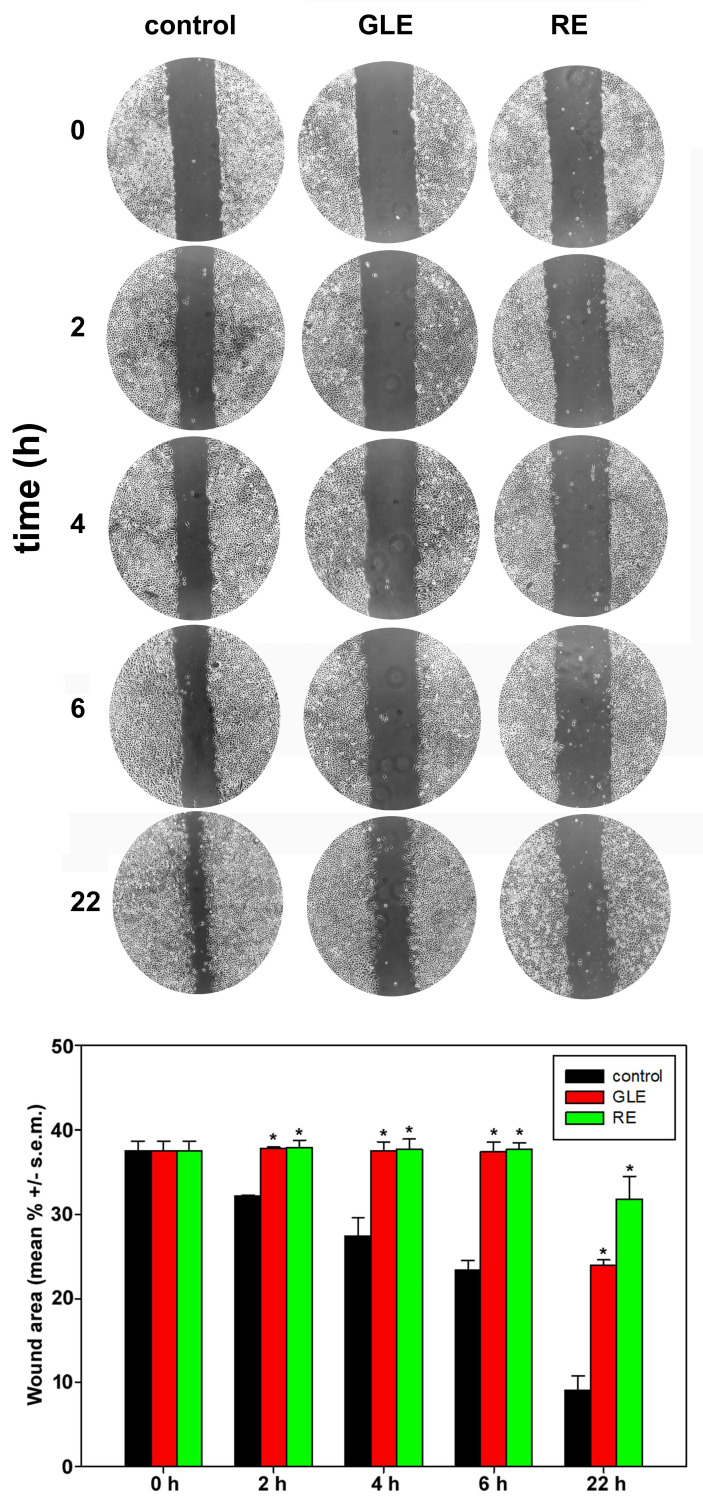
(Upper panel) representative phase contrast micrographs acquired during wound-healing experiments at different time intervals under control conditions and in the presence of GLE or RE at their IC_50_. The assay was performed in triplicate. Microscopic magnification = 20×. (Lower image) bar graph summarizing the data for wound closure by control and treated cells obtained from triplicate experiments. * normality test vs. control passed.

A scratch wound-healing assay was performed to examine the effect of the incubation with IC_50_24 of either GLE or RE on HepG2 cells’ locomotory ability. It is known that, in control conditions, HepG2 cells exhibit a motile attitude that allows them to drastically reduce the wounded area within 24 h [20,21,58,59]. In line with these indications, the panel of micrographs in Figure 12 shows that untreated HepG2 cells were endowed with a locomotory ability, resulting in the progressive decrease in the wound size (expressed as mean area % ± s.e.m): 37.5 ± 1.2 (0 h), 32.2 ± 0.1 (2 h), 27.4 ± 2.2 (4 h), 23.4 ± 1.1 (6 h), and 9.1 ± 1.7 (22 h) from the start of the experiment. Of note, it was acknowledged that the doubling time of HepG2 cells is less than 24 h [60,61]; conceivably, the effect here observed at smaller timeframes was not attributable to cell proliferation. On the other hand, HepG2 cells’ ability to migrate into the scratched zone was inhibited by their exposure to both extracts to a different extent. In particular, under both experimental conditions, the wound size was not modified within 6 h from the scratch. Mean area % ± s.e.m. in the presence of GLE and RE was 37.8 ± 0.2 and 37.9 ± 0.9 (2 h), 37.5 ± 1.1 and 37.7 ± 1.2 (4 h), and 37.4 ± 1.2 and 37.7 ± 0.8 (6 h) from the start of the experiment, respectively. After 22 h from the scratch, HepG2 cells cultured in the presence of GLE showed a moderate migratory attitude, determining the reduction of the gap area to 24 ± 0.6%, whereas, in the case of exposure to RE, the ability of cells to migrate into the denuded area appeared to be even lower and the wound size remained larger, its area being 31.8 ± 2.7%.

### 3.7. Potential Protein Contributors to the Observed GLE- and RE-Cytotoxic Activity 

In light of the cytotoxic role played by GLE and RE on HepG2 cells and the involvement, sometimes varying, of autophagy, apoptosis, imbalance of redox state, and derangement of mitochondrial function as emerged from the data previously shown, MS-based proteomic analyses were performed on the extracts after proteolysis of the samples. Overall, 100 proteins were identified by searching against the Alismatales database; this was necessary, as protein sequence databases for *P. oceanica* are highly incomplete. This bioinformatic similarity search identified 14 proteins contained in the preparations that might be potentially associated with the various aspects related to the impairment of the biological activities reported. The identity of the proteins and their relative semi-quantitative abundance in GLE and/or RE samples are reported in Table 3. A complete reference to the organismal databases used can be found as a Supplementary File (Table S1).

## 4. Discussion

The aquatic ecosystem constitutes the largest habitat of Earth, hosting an enormous diversity of animal and plant organisms and representing an underexploited source rich in natural bioactive molecules, which only to a small extent have been investigated for their potential utilization as treatment agents for numerous disease states, e.g., [62,63,64,65,66]. The present study was aimed to ascertain whether the water-soluble extracts from different parts of the seagrass *P. oceanica* grown in the northwestern Sicilian coastal area could exert any cytotoxic effect on HepG2 liver cancer cells (chosen as an in vitro model of a cancer histotype of the digestive system). The obtained data suggest the potential antitumoral role of the preparations from rhizomes and green leaves, whereas extracts from the beached brown leaves failed to show any effect on cell numbers at all the concentrations tested. On the other hand, although we showed that 24 h exposure to both RE and GLE decreased tumor cell numbers in a dose–response manner, with respect to the biological endpoints chosen, the two extracts appeared not to elicit fully overlapping effects.

Regarding the number and nature of the polyphenolic compounds found in the two different biologically active *Posidonia* matrices, they were mostly in agreement with the literature data [8,12,67]. The differences could be correlated with the extraction method followed, as well as the quantities extracted by volume/weight. Delphinidin-3-glucoside and quercetin 3-O-galactoside were the most abundant polyphenols in RE and lower amounts of vanillic acid and some procyanidin dimers were also above the quantitation-allowing threshold. Interestingly, Maciel et al. [68] reported that a low amount (5.39 μg/mL) of the delphinidin-3-glucoside-rich aqueous extract from *Hibiscus sabdariffa* acted as LC_50_ (50% lethal concentration) in HepG2 cells, whereas Martìnez-Alonso et al. [69] showed that the delphinidin-rich red bean extract acted by reducing ROS production. In addition, quercetin 3-O-galactoside (also known as hyperoside) was reported to arrest the HepG2 cell cycle by down-regulating the BMP-7/PI3K/AKT signaling [70]. Akbar et al. [71] demonstrated a substantial inhibition of HepG2 cell viability by vanillic acid, which induced cell-cycle perturbation **via** inhibition of cyclin-dependent kinase 2. Additionally, procyanidin B2 and B3 showed antiproliferative activity against HepG2 cells, the first being a proven cell-cycle inhibitor and apoptosis promoter [72,73]. Caffeic acid methyl ester was the most copious polyphenol in GLE and its cytotoxic activity against HepG2 cells was demonstrated by Zhou et al. [74]. In addition, according to the data of Razali et al. [75], it could be responsible for the sustained down-regulation of ROS observed during GLE treatment. Furthermore, the reduced dissipation of MMP observed after exposure of HepG2 cells to GLE may be conceivably due to the acknowledged protective effect of the compound towards mitochondrial dysfunctions in this cell line [76]. However, we cannot exclude the cooperation of other trace components of both GLE and RE in inducing the effects observed in our assays on HepG2 cells.

In the search for other molecular constituents that could be conceivably involved in the described cytotoxic activity, we performed a proteomic analysis of GLE and RE, which predicted a set of protein signatures putatively responsible for the observed panel of biological implications. Additionally, this component showed a certain degree of heterogeneity between the two preparations. Among the signatures identified, some can be associated with autophagy down-regulation. In fact, hepatocytic autophagy is known to be suppressed by adenosine kinase-mediated AMP formation and leucyl aminopeptidase over-expression [77,78]; acyl-CoA binding (ACB) protein has also been described as a suppressor of autophagy in breast cancer cells via its binding ability to phosphatidylethanolamine of the phagophore membrane, resulting in the inhibition of LC3 lipidation [79]. Other protein signatures may be mainly associated with the onset of apoptosis, also linked to the impairment of mitochondrial function and modulation of the autophagic flux. Zheng et al. [80] demonstrated the cell-cycle inhibitory- and apoptosis-triggering effect of phosphoglucomutase over-expression on colorectal tumor cells via the PI3K/AKT pathway. Human CutA1 protein was proven to sensitize HeLa cells to copper-induced inhibition of cell proliferation and promotion of apoptosis [81]; the mechanosensitive ion channel proteins were reported to induce mitochondrial dysfunctions that lead to autophagy reduction and apoptosis intensification [82]. Adenosylhomocysteinase is known to promote adenosine-induced apoptosis in HepG2 and esophageal cancer cells, also blocking the motility of the latter, and to inhibit autophagy in osteosarcoma cells in an MTORC1-independent manner [83,84,85]. In the mitochondria, the nucleoside-diphosphate kinase NME4 participates in the support of cardiolipin externalization following organelle dysfunction, thereby giving a signal for apoptosis [86]. Finally, a group of proteins shows antioxidant properties. High ROS scavenging ability has been found for recombinant 2-Cys peroxiredoxin from *Citrus sinensis* in Vero cells, heterologous ferredoxin-NADP reductase in Cos-7 cells, and recombinant peptidylprolyl cis-trans isomerase from *Pyropia yezoensis* in HepG2 cells [87,88,89]. On the other hand, the protective effect of glutathione reductase, glutathione transferase, and superoxide dismutase against oxidative damages is well-known [90,91]. Interestingly, Aniya and Imaizumi [92] reported that mitochondrial membrane-bound glutathione transferase may form a permeability transition pore, which leads to the loss of MMP. Due to the selective presence of this protein in RE, it might be conceivably responsible for the larger population of cells exhibiting mitochondrial depolarization following exposure to this extract. At present, we have no data confirming whether these proteins present in the aqueous samples can be effectively internalized by the cells and can exert their putative functions, apart from peptidyl-prolyl cis-trans isomerase that has been successfully administered as an antioxidant to HepG2 cells and superoxide dismutase that, along with its conjugates and mimetics, possesses an acknowledged significant therapeutic action/potential against several diseases in humans and animals [88,91,93]. On the other hand, it is worth-mentioning that we [94] have recently demonstrated that an isolated peptide obtained from RE shows an apoptotic-promoting ability on HepG2 cells, thus providing a first direct indication of the contribution of the protein component of the extract to the cytotoxic effect.

Exposure to both extracts were found to already down-regulate ROS accumulation at 4h of exposure, whereas after 24 h, although ROS generation induced by RE on individual cells was comparable to that of the control, the fraction of ROS^+^ cells was drastically reduced within the whole population. The decrease in intracellular ROS levels has been documented in quercetin-treated HepG2 cells by Jeon et al. [95] and linked to the anti-proliferative effect of the compound. The apoptotic-promoting role of ROS down-regulation in HepG2 cells was explained by Liu et al. [96], in terms of the lack of the required amount of an important class of redox-active signaling molecules necessary for cell survival and growth.

Autophagy is an important pathway that plays a key role in normal physiological processes, under several stressful conditions, i.e., in the presence of damaged organelles or aggregated misfolded proteins, and in conditions of nutrient deprivation. The modulation of autophagy plays dual roles in tumor suppression and promotion in many cancers. Much evidence supports autophagy modulation as a promising and potential therapeutic target [97,98,99]. Elevated levels of basal autophagy, such as those observed in HepG2 cells, enable cancer-cell survival, growth, and motility by fulfilling the high metabolic and energetic demands [100]. Thus, the suppression of “protective” autophagy may be one of the mechanisms of cytotoxicity of the extracts under study that leads to the inhibition of cell proliferation and the promotion of apoptosis. Interestingly, the reversion of autophagy inhibition by rapamycin was sufficient to restore cell viability in the presence of GLE, at which autophagy down-regulation as well as MMP dissipation and decrease in clonogenic potential was milder than following RE treatment, thus suggesting, in the latter case, a broader and multitarget death-triggering cell damage than mere autophagy inhibition. The more pronounced derangement of HepG2 cells’ healthy state by RE also appeared from the stronger inhibition of cell motility, as observed in the wound-healing assays, thereby suggesting that this preparation may be a potential powerful suppressor of the metastatic attitude of liver cancer cells.

At the molecular level, the data reported here show that autophagy in HepG2 cells showed an evident modulation, especially from 14 to 24 h of exposure and with greater effect for RE- than GLE-treated cells. However, in both cases there was a reduction in both the early and late autophagic processes, monitored by the accumulation of Beclin-1 and LC3-II/LC3-I ratio, respectively. On the other hand, during the early stages of treatment (4 h), the levels of autophagy seemed more evident in the treated sample than in the control, but this was recorded only for the late autophagy process (LC3-II/LC3-I ratio) and not for the early process (quantitation of Beclin-1). This finding could be justified, considering that LC3-II marker levels do not depend directly on its translation but on a post-translation modification, i.e., the rapid conversion of LC3-I to LC3-II through a lipidation event. Therefore, in the early times of exposure, the cells already appeared to perceive the effect of the treatments, transiently triggering a defense mechanism represented by the rapid conversion of LC3. At the same time, the levels of AVOs were reduced in this early phase. As is known, by promoting the digestion of the autophagosome content, the same LC3-II protein starts to be degraded in these acidic vesicles. Indeed, since there was a reduction in AVOs after both treatments, and therefore a reduction in the number of autophagosomes that fuse with lysosomes, the autophagic organelles persisted with their intact membrane marker (LC3-II). A reduction in LC3-II/LC3-I ratio levels was observed in treated cells after 14 and 24 h of exposure (always with greater effect for RE than GLE treatment), when the levels of AVOs were very low. Thus, this decline of the autophagic process could be attributed to the direct action of the treatments on the expression of the autophagic proteins. These data were confirmed by the levels of Beclin-1 protein in the Western blot assays, being the signals were always very low in the treated samples compared with the control samples and showed a more marked effect for RE- than GLE-treated cells. Looking at proteins destined for autophagic degradation through the analysis of the marker p62/SQSTM1, it could be noted that, after 14 h, the GLE-treated cells exhibited a peak of this marker, indicating the possible activation of a cytoprotective mechanism. This result was confirmed by the peak for hsp60 detected after this treatment, confirming that a probable cytoprotection was active against the accumulation of damaged proteins. After 4 h of treatment, the levels of hsp60 detected in both treatments were low compared with the control, indicating a possible early reduction in stress due to the clearance process promoted by autophagy (high LC3-II/LC3-I ratio levels).

However, in general, there was a reduction in the autophagic processes for longer treatment times and this was to be correlated with the simultaneous activation of apoptotic processes, which were marked after 24 h of exposure. At this stage, in fact, the levels of p62/SQSTM1 protein were high in GLE- and especially RE-treated cells, indicative of the accumulation of protein aggregates that the cells were unable to eliminate due to the suspension of the autophagic clearance process and the simultaneous triggering of apoptotic cell death. In this context, as expected, a parallel reduction in the cytoprotective mechanisms exerted by hsp60 was observed, thereby confirming a synergistic effect of the treatments that reduce defense strategies, i.e., autophagy and cytoprotective mechanisms towards stress, and increase cell death processes. Taken together, these data suggest that cells exposed to the extracts initially promote defense strategies, whereas they seem to offer less resistance to the persistence of the exposure and reduce the cell survival processes (autophagy) and the cytoprotection, giving way to the degenerative processes.

We have explored the cell response following exposure to the extracts by studying the activation levels of caspases—key mediators of apoptosis—in order to also identify a possible relationship with other survival or cell-death mechanisms. It is known that there is a crosstalk between autophagy and apoptosis in cancer [57] and that caspases can be involved in autophagy [101]. Among the initiator caspases, we did not detect the increase of signals related to caspase-8 and -9 (indicative of the activation of the extrinsic and intrinsic pathway, respectively), but the presence of cleaved caspase-1 and -2 was observed after 24 h for both extracts. Of note, caspase-1 cleavage was proven to match the up-regulation of the apoptosis rate recorded in xanthoangelol-treated HepG2 cells [102] and, similar to what we observed for RE- and GLE-treated cells, an increase in its activation in hepatocytes was paralleled to autophagy down-regulation, as shown by the loss of LC3 and Beclin-1 [103]. Caspase-2 possesses features of both initiator and effector caspases [104] and has been found as the sole activated caspase involved in the onset of apoptosis following exposure of HepG2 cells to TNF-α [105]. In general, its activity is linked to ROS scavenging and negative regulation of autophagy [106,107]. Our data are in line with these findings, since, under the experimental conditions used, we observed a decrease in ROS accumulation and ROS+ cell population and the down-regulation of AVOs and the autophagic markers LC3-II and Beclin-1, the latter especially in RE-treated cell samples.

Among the executioner caspases, we observed a marked increase in the signals of cleaved caspase-3 after 24 h of treatment with GLE and a significative increase in those of activated caspase-6 for both treatments compared with the controls. Apoptosis of HepG2 cells after bile acid treatments was reported to be caspase-6 dependent, whereas caspase-3 activation was found to be responsible for the apoptotic death of HepG2 cells following exposure to sulforaphane and ursolic acid, in the latter case due to the inactivation of the PI3K/Akt/survivin pathway; moreover, caspase-3 was proven to inhibit the generation of ROS, similar to what was prominently observed in our experimental system in the presence of GLE. These two caspases have also been placed either upstream or downstream of each other in different experimental systems, thereby suggesting the existence of a complex pattern of mutual interactions [108,109,110]. Additionally, in this case, we found a correlation with the autophagic status observed in our assays for exposed cells. In fact, as reported in the literature, several pro-apoptotic caspases directly interact with and cleave essential ATG proteins, resulting in the inhibition of autophagy; for example, caspase-3 cleaves Beclin-1 and inhibits autophagy [54]. On the other hand, caspase-6 has a role in cleaving p62, and p62 N-terminal caspase-6 cleavage product (p62-N) plays a dominant negative role to block p62-droplet formation and autophagosome formation [111], confirming an autophagy modulation through the caspase-6—p62 axis under certain stress stimuli. Similarly, this was also observable in our experiments, where, considering the entire treatment, the lowest levels of p62 were detected after 24 h of exposure, notably in the presence of GLE.

The results obtained on caspase activities, taken together, allow us to assume that, in the response to the treatments on HepG2 cells, a crosstalk between autophagy and apoptosis exists, suggesting that such a response is modulated through the orchestration of these processes.

## 5. Conclusions

In conclusion, we have demonstrated the cytotoxicity of aqueous extracts from different parts of the seagrass *P. oceanica* in a liver cancer cell model system, identifying death-promoting mechanisms that involve the modulation of autophagy, apoptosis, and cell redox status and the impairment of MMP, although partially differentiating conceivably due to the different compositions of GLE and RE. Hence, these marine-derived natural materials are worth further exploration addressing the more detailed study of the biological properties of the phytocomplexes intended to develop novel alternative agents for prevention and/or treatment of liver tumors and beneficial supplements for the formulation of functional food and food-packaging materials endowed with antioxidant and anticancer properties.

## Figures and Tables

**Table 1:** Primers used for PCR amplification.

Gene (Primer)	Sequence (5′→ 3′)	Reference
*BAD* (sense)	GTTCCAGATCCCAGAGTTTGAGC	[31]
*BAD* (antisense)	TTAAAGGAGTCCACAAACTCGTCACT	
*BAX* (sense)	ATGGACGGTCCGGGGAGCAGC	[31]
*BAX* (antisense)	CCCCAGTTGAAGTTGCCGTCAG	
*BCL2* (sense)	GCCTTTGTGGAACTGTACGGC	[31]
*BCL2* (antisense)	GGCAGTAAATAGCTGATTCGACGTT	
*DAPK* (sense)	GATGGCAACATGCCTATCGTG	[31]
*DAPK* (antisense)	GATGAAGAGTCCTCGGTGCGTAT	
*JUN* (sense)	CCCCAAGATCCTGAAACAGA	[32]
*JUN* (antisense)	CCGTTGCTGGACTGGATTAT	
*FOS* (sense)	CCAACTTTATCCCCACGGTGAC	[33]
*FOS* (antisense)	TGGCAATCTCGGTCTGCAAC	
*ACTB* (sense)	GGAAGGTGGACAGCGAGGCC	[34]
*ACTB* (antisense)	GTGACGTGGACATCCGCAAAG	

**Table 2:** Quali-quantitative determination of the phenolic components of GLE, BLE, and RE. n.q.—not quantifiable.

Polyphenol	GLE (μg/g)	BLE (μg/g)	RE (μg/g)
Delphinidin-3-glucoside	n.q	-	11.52
Quercetin 3-O-galactoside	n.q	-	10.81
Procyanidin dimer B type isomer 2	n.q	-	0.20
Procyanidin dimer B type isomer 3	n.q	n.q	0.30
Pro-Cyanidin-Dimer-B	n.q	n.q	-
Cyanidin 3-O-glucoside	-	n.q	-
Vanillic acid	-	-	0.6
Gallic acid	n.q	-	n.q.
Kaempferol 3-O-glucoside	-	-	n.q.
Kaempferol	n.q	-	-
Kaempferol 7-O-hexuronide	-	-	n.q.
Procyanidin trimer B type	-	-	n.q.
Gallic acid ethyl ester	-	-	n.q.
Catechin	n.q	n.q	n.q.
Epicatechin	-	-	n.q.
Myricetin	-	-	n.q.
Peonidin 3-O-hexoside isomer	-	-	n.q.
Malvidin 3-O-pentoside	-	-	n.q.
Quercetin 3-O-hexuronide	-	-	n.q.
Quercetin 3-O-(6”-malonyl) hexoside	n.q	n.q	-
Resveratrol tetramer	-	-	n.q.
Caffeic acid methyl ester	0.37	-	-
Caffeic acid	n.q	n.q	-
p-Coumaric acid	n.q.	-	-
Ellagic acid	n.q	-	-
p-Hydroxybenzoic acid	-	0.29	-
Ferulic acid	-	n.q	-
Myricetin	-	n.q	-
Myricetin 3-O-hexoside	-	n.q	-
Petunidin 3-O-(6”-acetyl) hexoside	-	n.q	-

**Table 3:** Cytotoxic activity-associated proteins identified in GLE and RE.

Accession Number/ Protein Description	GLE Amount	RE Amount
A0A843WDB0 ACB domain-containing protein	5.34 × 10^4^	3.72 × 10^5^
A0A1D1ZBB9 Adenosine kinase (Fragment)	4.68 × 10^4^	3.92 × 10^4^
A0A1D1YQU6 Adenosylhomocysteinase (Fragment) A0A1D1YZX9 2-Cys peroxiredoxin BAS1-like, chloroplastic (Fragment) A0A0K9PQ40 Ferredoxin-NADP reductase, chloroplastic A0A1D1ZGY1 Glutathione reductase A0A0K9P699 Glutathione transferase A0A0K9P881 Leucyl aminopeptidase A0A843XPL2 Mechanosensitive ion channel protein A0A0K9Q3S1 Nucleoside-diphosphate kinase A0A0K9P7Q7 Peptidyl-prolyl cis-trans isomerase F8U875 Phosphoglucomutase (alpha-D-glucose- 1,6-bisphosphate-dependent) A0A0K9PHA0 Protein CutA 1, chloroplastic A0A1D1XUN0 Superoxide dismutase	1.06 × 10^5^ 5850 1.37 × 10^5^ 0 0 9.56 × 10^4^ 7.32 × 10^6^ 3.87 × 10^4^ 0 0 0 3.92 × 10^5^	6.39 × 10^5^ 6.58 × 10^4^ 0 1.31 × 10^5^ 2.01 × 10^5^ 4.23 × 10^4^ 5.62 × 10^6^ 2.31 × 10^5^ 1.96 × 10^5^ 1.22× 10^5^ 4.93 × 10^5^ 1.95× 10^6^

## Data Availability

The data presented in the current study are available from the corresponding author upon request.

## Supplementary Materials

The following supporting information can be downloaded at: https://www.mdpi.com/article/10.3390/biology12040616/s1.

## References

[1] Bellissimo G., Sirchia B., Ruvolo V., Carboni D., De Vincenzi M., Bonora L. (2020). Monitoring of *Posidonia oceanica* meadows in the Sicilian coasts under the Water Framework Directive (WFD). Eighth International Symposium “Monitoring of Mediterranean Coastal Areas. Problems and Measurement Techniques”.

[2] Bernard P., Pesando D. (1989). Antibacterial and Antifungal Activity of Extracts from the Rhizomes of the Mediterranean Seagrass *Posidonia oceanica* (L.) Delile. Bot. Mar..

[3] Berfad M.A., Alnour T.M.S. (2014). Phytochemical analysis and Antibacterial activity of the 5 different extract from the seagrasses *Posidonia oceanica*. J. Med. Plants Stud..

[4] Gokce G., Haznedaroglu M.Z. (2008). Evaluation of antidiabetic, antioxidant and vasoprotective effects of *Posidonia oceanica* extract. J. Ethnopharmacol..

[5] Cornara L., Pastorino G., Borghesi B., Salis A., Clericuzio M., Marchetti C., Damonte G., Burlando B. (2018). *Posidonia oceanica* (L.) Delile Ethanolic Extract Modulates Cell Activities with Skin Health Applications. Mar. Drugs.

[6] Vasarri M., Leri M., Barletta E., Ramazzotti M., Marzocchini R., Degl’Innocenti D. (2020). Anti-inflammatory properties of the marine plant *Posidonia oceanica* (L.) Delile. J. Ethnopharmacol..

[7] Vasarri M., Barletta E., Ramazzotti M., Degl’Innocenti D. (2020). In vitro anti-glycation activity of the marine plant *Posidonia oceanica* (L.) Delile. J. Ethnopharmacol..

[8] Messina C.M., Arena R., Manuguerra S., Pericot Y., Curcuraci E., Kerninon F., Renda G., Hellio C., Santulli A. (2021). Antioxidant Bioactivity of Extracts from Beach Cast Leaves of *Posidonia oceanica* (L.) Delile. Mar. Drugs.

[9] Barletta E., Ramazzotti M., Fratianni F., Pessani D., Degl’Innocenti D. (2015). Hydrophilic extract from *Posidonia oceanica* inhibits activity and expression of gelatinases and prevents HT1080 human fibrosarcoma cell line invasion. Cell Adhes. Migr..

[10] Leri M., Ramazzotti M., Vasarri M., Peri S., Barletta E., Pretti C., Degl’Innocenti D. (2018). Bioactive Compounds from *Posidonia oceanica* (L.) Delile Impair Malignant Cell Migration through Autophagy Modulation. Mar. Drugs.

[11] Vasarri M., Leri M., Barletta E., Pretti C., Degl’Innocenti D. (2021). *Posidonia oceanica* (L.) Delile Dampens Cell Migration of Human Neuroblastoma Cells. Mar. Drugs.

[12] Farid M.M., Marzouk M.M., Hussein S.R., Elkhateeb A., Abdel-Hameed E.S. (2018). Comparative study of *Posidonia oceanica* L.: LC/ESI/MS analysis, cytotoxic activity and chemosystematic significance. J. Mater. Environ. Sci..

[13] Asafo-Agyei K.O., Samant H. (2022). Hepatocellular Carcinoma. StatPearls [Internet].

[14] Philips C.A., Rajesh S., Nair D.C., Ahamed R., Abduljaleel J.K., Augustine P. (2021). Hepatocellular Carcinoma in 2021: An Exhaustive Update. Cureus.

[15] Brar T.S., Hilgenfeldt E., Soldevila-Pico C., Liu C. (2018). Etiology and Pathogenesis of Hepatocellular Carcinoma. Precision Molecular Pathology of Liver Cancer. Molecular Pathology Library.

[16] Farzaneh Z., Vosough M., Agarwal T., Farzaneh M. (2021). Critical signaling pathways governing hepatocellular carcinoma behavior; small molecule-based approaches. Cancer Cell Int..

[17] Kaal J., Serrano O., Nieropd K.G.J., Schellekense J., Martínez Cortizas A., Mateo M.A. (2016). Molecular composition of plant parts and sediment organic matter in a Mediterranean seagrass (*Posidonia oceanica*) mat. Aquat. Bot..

[18] Astudillo-Pascual M., Domínguez I., Aguilera P.A., Garrido Frenich A. (2021). New Phenolic Compounds in *Posidonia oceanica* Seagrass: A Comprehensive Array Using High Resolution Mass Spectrometry. Plants.

[19] Donato M.T., Tolosa L., Gómez-Lechón M.J. (2015). Culture and Functional Characterization of Human Hepatoma HepG2 Cells. Meth. Mol. Biol..

[20] Luparello C., Branni R., Abruscato G., Lazzara V., Drahos L., Arizza V., Mauro M., Di Stefano V., Vazzana M. (2022). Cytotoxic capability and the associated proteomic profile of cell-free coelomic fluid extracts from the edible sea cucumber *Holothuria tubulosa* on HepG2 liver cancer cells. EXCLI J..

[21] Luparello C., Branni R., Abruscato G., Lazzara V., Sugár S., Arizza V., Mauro M., Di Stefano V., Vazzana M. (2022). Biological and Proteomic Characterization of the Anti-Cancer Potency of Aqueous Extracts from Cell-Free Coelomic Fluid of *Arbacia lixula* Sea Urchin in an In Vitro Model of Human Hepatocellular Carcinoma. J. Mar. Sci. Eng..

[22] Strober W. (2015). Trypan Blue Exclusion Test of Cell Viability. Curr. Protoc. Immunol..

[23] Chou T.C., Martin N. (2004). CompuSyn for Drug Combination User’s Guide: A Computer Program for Quantitation of Synergism and Antagonism in Drug Combinations, and the Determination of IC50 and ED50 Values. https://combosyn.com/uat/pdf/CompuSyn_users_guide.pdf.

[24] Librizzi M., Longo A., Chiarelli R., Amin J., Spencer J., Luparello C. (2012). Cytotoxic effects of Jay Amin hydroxamic acid (JAHA), a ferrocene-based class I histone deacetylase inhibitor, on triple-negative MDA-MB231 breast cancer cells. Chem. Res. Toxicol..

[25] Ramos A.A., Prata-Sena M., Castro-Carvalho B., Dethoup T., Buttachon S., Kijjoa A., Rocha E. (2015). Potential of four marine-derived fungi extracts as anti-proliferative and cell death-inducing agents in seven human cancer cell lines. Asian Pac. J. Trop. Med..

[26] Luparello C., Asaro D.M.L., Cruciata I., Hassell-Hart S., Sansook S., Spencer J., Caradonna F. (2019). Cytotoxic activity of the histone deacetylase 3-selective inhibitor Pojamide on MDA-MB-231 triple-negative breast cancer cells. Int. J. Mol. Sci..

[27] Luparello C., Ragona D., Asaro D.M.L., Lazzara V., Affranchi F., Celi M., Arizza V., Vazzana M. (2019). Cytotoxic Potential of the Coelomic Fluid Extracted from the Sea Cucumber *Holothuria tubulosa* against Triple-Negative MDA-MB231 Breast Cancer Cells. Biology.

[28] Luparello C., Ragona D., Asaro D.M.L., Lazzara V., Affranchi F., Arizza V., Vazzana M. (2020). Cell-Free Coelomic Fluid Extracts of the Sea Urchin *Arbacia lixula* Impair Mitochondrial Potential and Cell Cycle Distribution and Stimulate Reactive Oxygen Species Production and Autophagic Activity in Triple-Negative MDA-MB231 Breast Cancer Cells. J. Mar.Sci. Eng..

[29] Chiarelli R., Martino C., Roccheri M.C., Cancemi P. (2021). Toxic effects induced by vanadium on sea urchin embryos. Chemosphere.

[30] Aranda P.S., LaJoie D.M., Jorcyk C.L. (2012). Bleach gel: A simple agarose gel for analyzing RNA quality. Electrophoresis.

[31] Luparello C., Sirchia R., Lo Sasso B. (2008). Midregion PTHrP regulates Rip1 and caspase expression in MDA-MB231 breast cancer cells. Breast Cancer Res. Treat..

[32] Luparello C., Longo A., Vetrano M. (2012). Exposure to cadmium chloride influences astrocyte-elevated gene-1 (AEG-1) expression in MDA-MB231 human breast cancer cells. Biochimie.

[33] Ronchetti S.A., Miler E.A., Duvilanski B.H., Cabilla J.P. (2013). Cadmium mimics estrogen-driven cell proliferation and prolactin secretion from anterior pituitary cells. PLoS ONE.

[34] Qi M., Zhou Y., Liu J., Ou X., Li M., Long X., Ye J., Yu G. (2018). AngII induces HepG2 cells to activate epithelial-mesenchymal transition. Exp. Ther. Med..

[35] Franken N.A., Rodermond H.M., Stap J., Haveman J., van Bree C. (2006). Clonogenic assay of cells in vitro. Nat. Protoc..

[36] Chao X., Zhou X., Zheng G., Dong C., Zhang W., Song X., Jin T. (2014). Osthole induces G_2_/M cell cycle arrest and apoptosis in human hepatocellular carcinoma HepG2 cells. Pharm. Biol..

[37] Li Q., He Z., Liu J., Wu J., Tan G., Jiang J., Su Z., Cao M. (2019). *Paris polyphylla* 26 triggers G_2_/M phase arrest and induces apoptosis in HepG2 cells via inhibition of the Akt signaling pathway. J. Int. Med. Res..

[38] Golestani Eimani B., Sanati M.H., Houshmand M., Ataei M., Akbarian F., Shakhssalim N. (2014). Expression and prognostic significance of bcl-2 and bax in the progression and clinical outcome of transitional bladder cell carcinoma. Cell J..

[39] Kale J., Osterlund E., Andrews D. (2018). BCL-2 family proteins: Changing partners in the dance towards death. Cell Death Differ..

[40] Singh R., Czaja M.J. (2007). Regulation of hepatocyte apoptosis by oxidative stress. J. Gastroenterol. Hepatol..

[41] Podar K., Raab M.S., Tonon G., Sattler M., Barilà D., Zhang J., Tai Y.T., Yasui H., Raje N., DePinho R.A. (2007). Up-regulation of c-Jun inhibits proliferation and induces apoptosis via caspase-triggered c-Abl cleavage in human multiple myeloma. Cancer Res..

[42] Yuan Z., Gong S., Luo J., Zheng Z., Song B., Ma S., Guo J., Hu C., Thiel G., Vinson C. (2009). Opposing roles for ATF2 and c-Fos in c-Jun-mediated neuronal apoptosis. Mol. Cell. Biol..

[43] Gao F., Zhou L., Li M., Liu W., Yang S., Li W. (2020). Inhibition of ERKs/Akt-Mediated c-Fos Expression Is Required for Piperlongumine-Induced Cyclin D1 Downregulation and Tumor Suppression in Colorectal Cancer Cells. Onco Targets Ther..

[44] Yang C.W., Lee Y.Z., Hsu H.Y., Wu C.M., Chang H.Y., Chao Y.S., Lee S.J. (2013). c-Jun-mediated anticancer mechanisms of tylophorine. Carcinogenesis.

[45] Schlossberg H., Zhang Y., Dudus L., Engelhardt J.F. (1996). Expression of *c-fos* and *c-jun* during hepatocellular remodeling following ischemia/reperfusion in mouse liver. Hepatology.

[46] Auten R., Davis J. (2009). Oxygen Toxicity and Reactive Oxygen Species: The Devil is in the Details. Pediatr. Res..

[47] Zaidieh T., Smith J.R., Ball K.E., An Q. (2019). ROS as a novel indicator to predict anticancer drug efficacy. BMC Cancer.

[48] Jiang J., Briedé J.J., Jennen D.G., Van Summeren A., Saritas-Brauers K., Schaart G., Kleinjans J.C., de Kok T.M. (2015). Increased mitochondrial ROS formation by acetaminophen in human hepatic cells is associated with gene expression changes suggesting disruption of the mitochondrial electron transport chain. Toxicol. Lett..

[49] Belyaeva E.A., Dymkowska D., Wieckowski M.R., Wojtczak L. (2008). Mitochondria as an important target in heavy metal toxicity in rat hepatoma AS-30D cells. Toxicol. Appl. Pharmacol..

[50] Kenny T.C., Craig A.J., Villanueva A., Germain D. (2019). Mitohormesis Primes Tumor Invasion and Metastasis. Cell Rep.

[51] Bjørkøy G., Lamark T., Johansen T. (2006). p62/SQSTM1: A missing link between protein aggregates and the autophagy machinery. Autophagy.

[52] Decuypere J.P., Parys J.B., Bultynck G. (2012). Regulation of the autophagic bcl-2/beclin 1 interaction. Cells.

[53] Pace A., Barone G., Lauria A., Martorana A., Piccionello A.P., Pierro P., Terenzi A., Almerico A.M., Buscemi S., Campanella C. (2013). Hsp60, a novel target for antitumor therapy: Structure-function features and prospective drugs design. Curr. Pharm. Des..

[54] Klionsky D.J., Abdel-Aziz A.K., Abdelfatah S., Abdellatif M., Abdoli A., Abel S., Abeliovich H., Abildgaard M.H., Abudu Y.P., Acevedo-Arozena A. (2021). Guidelines for the use and interpretation of assays for monitoring autophagy (4th edition). Autophagy.

[55] Singh P., Ravanan P., Talwar P. (2016). Death Associated Protein Kinase 1 (DAPK1): A Regulator of Apoptosis and Autophagy. Front. Mol. Neurosci..

[56] Farag A.K., Roh E.J. (2019). Death-associated protein kinase (DAPK) family modulators: Current and future therapeutic outcomes. Med. Res. Rev..

[57] Yogev O., Goldberg R., Anzi S., Yogev O., Shaulian E. (2010). Jun proteins are starvation-regulated inhibitors of autophagy. Cancer Res..

[58] Xie X., Zhu H., Zhang J., Wang M., Zhu L., Guo Z., Shen W., Wang D. (2017). Solamargine inhibits the migration and invasion of HepG2 cells by blocking epithelial-to-mesenchymal transition. Oncol. Lett..

[59] Zheng J., Shao Y., Jiang Y., Chen F., Liu S., Yu N., Zhang D., Liu X., Zou L. (2019). Tangeretin inhibits hepatocellular carcinoma proliferation and migration by promoting autophagy-related BECLIN1. Cancer Manag. Res..

[60] Furth E.E., Sprecher H., Fisher E.A., Fleishman H.D., Laposata M. (1992). An in vitro model for essential fatty acid deficiency: HepG2 cells permanently maintained in lipid-free medium. J. Lipid Res..

[61] Desquiret V., Loiseau D., Jacques C., Douay O., Malthièry Y., Ritz P., Roussel D. (2006). Dinitrophenol-induced mitochondrial uncoupling in vivo triggers respiratory adaptation in HepG2 cells. Biochim. Biophys. Acta.

[62] Lazzara V., Arizza V., Luparello C., Mauro M., Vazzana M. (2019). Bright Spots in The Darkness of Cancer: A Review of Starfishes-Derived Compounds and Their Anti-Tumor Action. Mar. Drugs.

[63] Luparello C., Mauro M., Lazzara V., Vazzana M. (2020). Collective Locomotion of Human Cells, Wound Healing and Their Control by Extracts and Isolated Compounds from Marine Invertebrates. Molecules.

[64] Luparello C., Mauro M., Arizza V., Vazzana M. (2020). Histone Deacetylase Inhibitors from Marine Invertebrates. Biology.

[65] Mauro M., Lazzara V., Punginelli D., Arizza V., Vazzana M. (2020). Antitumoral compounds from vertebrate sister group: A review of Mediterranean ascidians. Dev. Comp. Immunol..

[66] Punginelli D., Schillaci D., Mauro M., Deidun A., Barone G., Arizza V., Vazzana M. (2022). The potential of antimicrobial peptides isolated from freshwater crayfish species in new drug development: A review. Dev. Comp. Immunol..

[67] Ammar N.M., Hassan H.A., Mohammed M.A., Serag A., Abd El-Alim S.H., Elmotasem H., El Raey M., El Gendy A.N., Sobeh M., Abdel-Hamid A.Z. (2021). Metabolomic profiling to reveal the therapeutic potency of *Posidonia oceanica* nanoparticles in diabetic rats. RSC Adv..

[68] Maciel L.G., do Carmo M.A.V., Azevedo L., Daguer H., Molognoni L., de Almeida M.M., Granato D., Rosso N.D. (2018). *Hibiscus sabdariffa* anthocyanins-rich extract: Chemical stability, in vitro antioxidant and antiproliferative activities. Food Chem. Toxicol..

[69] Martínez-Alonso C., Taroncher M., Castaldo L., Izzo L., Rodríguez-Carrasco Y., Ritieni A., Ruiz M.J. (2022). Effect of Phenolic Extract from Red Beans (*Phaseolus vulgaris* L.) on T-2 Toxin-Induced Cytotoxicity in HepG2 Cells. Foods.

[70] Wei S., Sun Y., Wang L., Zhang T., Hu W., Bao W., Mao L., Chen J., Li H., Wen Y. (2021). Hyperoside suppresses BMP-7-dependent PI3K/AKT pathway in human hepatocellular carcinoma cells. Ann. Transl. Med..

[71] Akbar S., Ishtiaq S., Jahangir M., Elhady S.S., Bogari H.A., Alahdal A.M., Ashour M.L., Youssef F.S. (2021). Evaluation of The Antioxidant, Antimicrobial, and Anticancer Activities of *Dicliptera bupleuroides* Isolated Compounds Using In Vitro and In Silico Studies. Molecules.

[72] Ibrahim T.A., El Dib R.A., Al-Youssef H.M., Amina M. (2019). Chemical composition and antimicrobial and cytotoxic activities of *Antidesm abunius* L. Pak. J. Pharm. Sci..

[73] Huang H.M., Ho C.Y., Chang G.R., Shia W.Y., Lai C.H., Chao C.H., Wang C.M. (2021). HPLC/ESI-MS and NMR Analysis of Chemical Constitutes in Bioactive Extract from the Root Nodule of *Vaccinium emarginatum*. Pharmaceuticals.

[74] Zhou M.N., Liu P., Jing S.J., Sun M., Li X., Zhang W., Liu B. (2022). Chemical constituents of Scrophulariae Radix and their antitumor activities in vitro. Zhongguo Zhong Yao Za Zhi.

[75] Razali N., Mat Junit S., Ariffin A., Ramli N.S., Abdul Aziz A. (2015). Polyphenols from the extract and fraction of *T. indica* seeds protected HepG2 cells against oxidative stress. BMC Complement. Altern. Med..

[76] Tsai T.H., Yu C.H., Chang Y.P., Lin Y.T., Huang C.J., Kuo Y.H., Tsai P.J. (2017). Protective Effect of Caffeic Acid Derivatives on tert-Butyl Hydroperoxide-Induced Oxidative Hepato-Toxicity and Mitochondrial Dysfunction in HepG2 Cells. Molecules.

[77] Samari H.R., Seglen P.O. (1998). Inhibition of hepatocytic autophagy by adenosine, aminoimidazole-4-carboxamide riboside, and N6-mercaptopurine riboside. Evidence for involvement of amp-activated protein kinase. J. Biol. Chem..

[78] Feng L., Chen Y., Xu K., Li Y., Riaz F., Lu K., Chen Q., Du X., Wu L., Cao D. (2022). Cholesterol-induced leucine aminopeptidase 3 (LAP3) upregulation inhibits cell autophagy in pathogenesis of NAFLD. Aging.

[79] Udupa P., Kumar A., Parit R., Ghosh D.K. (2022). Acyl-CoA binding protein regulates nutrient-dependent autophagy. Metabolism.

[80] Zheng Z., Zhang X., Bai J., Long L., Liu D., Zhou Y. (2022). PGM1 suppresses colorectal cancer cell migration and invasion by regulating the PI3K/AKT pathway. Cancer Cell Int..

[81] Yang J., Li Q., Yang H., Yan L., Yang L., Yu L. (2008). Overexpression of human CUTA isoform2 enhances the cytotoxicity of copper to HeLa cells. Acta Biochim. Pol..

[82] Shi S., Kang X.J., Zhou Z., He Z.M., Zheng S., He S.S. (2022). Excessive mechanical stress-induced intervertebral disc degeneration is related to Piezo1 overexpression triggering the imbalance of autophagy/apoptosis in human nucleus pulpous. Arthritis Res. Ther..

[83] Hermes M., Osswald H., Kloor D. (2007). Role of S-adenosylhomocysteine hydrolase in adenosine-induced apoptosis in HepG2 cells. Exp. Cell Res..

[84] Li Q., Mao L., Wang R., Zhu L., Xue L. (2014). Overexpression of S-adenosylhomocysteine hydrolase (SAHH) in esophageal squamous cell carcinoma (ESCC) cell lines: Effects on apoptosis, migration and adhesion of cells. Mol. Biol. Rep..

[85] Huang W., Li N., Zhang Y., Wang X., Yin M., Lei Q.Y. (2022). AHCYL1 senses SAH to inhibit autophagy through interaction with PIK3C3 in an MTORC1-independent manner. Autophagy.

[86] Schlattner U., Tokarska-Schlattner M., Epand R.M., Boissan M., Lacombe M.L., Kagan V.E. (2018). NME4/nucleoside diphosphate kinase D in cardiolipin signaling and mitophagy. Lab. Investig..

[87] Mediavilla M.G., Di Venanzio G.A., Guibert E.E., Tiribelli C. (2010). Heterologous ferredoxin reductase and flavodoxin protect Cos-7 cells from oxidative stress. PLoS ONE.

[88] Kim E.Y., Choi Y.H., Choi C.G., Nam T.J. (2017). Effects of the cyclophilin-type peptidylprolyl cis-trans isomerase from *Pyropia yezoensis* against hydrogen peroxide-induced oxidative stress in HepG2 cells. Mol. Med. Rep..

[89] Gupta D.N., Rani R., Kokane A.D., Ghosh D.K., Tomar S., Sharma A.K. (2022). Characterization of a cytoplasmic 2-Cys peroxiredoxin from *Citrus sinensis* and its potential role in protection from oxidative damage and wound healing. Int. J. Biol. Macromol..

[90] Couto N., Wood J., Barber J. (2016). The role of glutathione reductase and related enzymes on cellular redox homoeostasis network. Free Radic Biol Med.

[91] Elhusseiny S.M., El-Mahdy T.S., Awad M.F., Elleboudy N.S., Farag M.M.S., Yassein M.A., Aboshanab K.M. (2021). Proteome Analysis and In Vitro Antiviral, Anticancer and Antioxidant Capacities of the Aqueous Extracts of *Lentinula edodes* and *Pleurotus ostreatus* Edible Mushrooms. Molecules.

[92] Aniya Y., Imaizumi N. (2011). Mitochondrial glutathione transferases involving a new function for membrane permeability transition pore regulation. Drug Metab. Rev..

[93] Younus H. (2018). Therapeutic potentials of superoxide dismutase. Int. J. Health Sci..

[94] Punginelli D., Catania V., Abruscato G., Luparello C., Vazzana M., Mauro M., Cunsolo V., Saletti R., Di Francesco A., Arizza V. (2023). New Bioactive Peptides from the Mediterranean Seagrass *Posidonia oceanica* (L.) Delile and Their Impact on Antimicrobial Activity and Apoptosis of Human Cancer Cells. Int. J. Mol. Sci..

[95] Jeon J.S., Kwon S., Ban K., Kwon Hong Y., Ahn C., Sung J.S., Choi I. (2018). Regulation of the Intracellular ROS Level Is Critical for the Antiproliferative Effect of Quercetin in the Hepatocellular Carcinoma Cell Line HepG2. Nutr. Cancer.

[96] Liu B., Tan X., Liang J., Wu S., Liu J., Zhang Q., Zhu R. (2014). A reduction in reactive oxygen species contributes to dihydromyricetin-induced apoptosis in human hepatocellular carcinoma cells. Sci. Rep..

[97] Mizushima N. (2007). Autophagy: Process and function. Genes Dev..

[98] Morel E., Mehrpour M., Botti J., Dupont N., Hamai A., Nascimbeni A.C., Codogno P. (2017). Autophagy: A Druggable Process. Annu. Rev. Pharmacol. Toxicol..

[99] Yun C.W., Lee S.H. (2018). The Roles of Autophagy in Cancer. Int. J. Mol. Sci..

[100] Luparello C. (2021). Marine Animal-Derived Compounds and Autophagy Modulation in Breast Cancer Cells. Foundations.

[101] Tsapras P., Nezis I.P. (2017). Caspase involvement in autophagy. Cell Death Differ..

[102] Pang X., Gao X., Liu F., Jiang Y., Wang M., Li Q., Li Z. (2021). Xanthoangelol modulates Caspase-1-dependent pyroptotic death among hepatocellular carcinoma cells with high expression of GSDMD. J. Funct. Foods.

[103] Sun Q., Fan J., Billiar T.R., Scott M.J. (2017). Inflammasome and autophagy regulation-a two-way street. Mol Med.

[104] Vigneswara V., Ahmed Z. (2020). The Role of Caspase-2 in Regulating Cell Fate. Cells.

[105] Tsagarakis N.J., Drygiannakis I., Batistakis A.G., Kolios G., Kouroumalis E.A. (2011). Octreotide induces caspase activation and apoptosis in human hepatoma HepG2 cells. World J. Gastroenterol..

[106] Shalini S., Dorstyn L., Wilson C., Puccini J., Ho L., Kumar S. (2012). Impaired antioxidant defence and accumulation of oxidative stress in caspase-2-deficient mice. Cell Death Differ..

[107] Tiwari M., Sharma L.K., Vanegas D., Callaway D.A., Bai Y., Lechleiter J.D., Herman B. (2014). A nonapoptotic role for CASP2/caspase 2: Modulation of autophagy. Autophagy.

[108] Park S.Y., Kim G.Y., Bae S.J., Yoo Y.H., Choi Y.H. (2007). Induction of apoptosis by isothiocyanate sulforaphane in human cervical carcinoma HeLa and hepatocarcinoma HepG2 cells through activation of caspase-3. Oncol. Rep..

[109] Rust C., Wild N., Bernt C., Vennegeerts T., Wimmer R., Beuers U. (2009). Bile acid-induced apoptosis in hepatocytes is caspase-6-dependent. J. Biol. Chem..

[110] Tang C., Lu Y.H., Xie J.H., Wang F., Zou J.N., Yang J.S., Xing Y.Y., Xi T. (2009). Downregulation of survivin and activation of caspase-3 through the PI3K/Akt pathway in ursolic acid-induced HepG2 cell apoptosis. Anticancer Drugs.

[111] Valionyte E., Yang Y., Griffiths S.A., Bone A.T., Barrow E.R., Sharma V., Lu B., Luo S. (2022). The caspase-6-p62 axis modulates p62 droplets based autophagy in a dominant-negative manner. Cell Death Differ..

