# Supplementary material for: In Vitro Cytotoxic Effect of Aqueous Extracts from Leaves and Rhizomes of the Seagrass Posidonia oceanica (L.) Delile on HepG2 Liver Cancer Cells: Focus on Autophagy and Apoptosis
Source: Biology (Basel). 2023 Apr 18;12(4):616. doi: 10.3390/biology12040616 (PMC10135731; doi:10.3390/biology12040616)
Supplement: Supplementary file 1 [file biology-12-00616-s001.zip › biology-2303112-supplementary.pdf]

Figure S1

Original images of the Western blots for Hsp60 and LC3

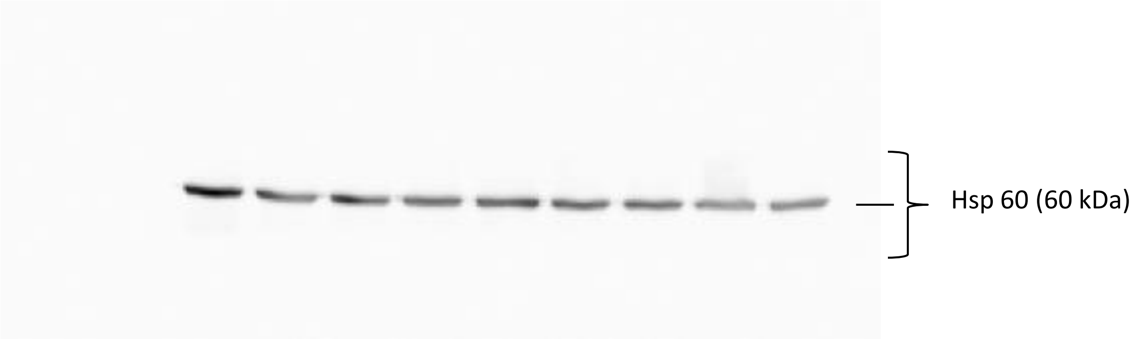

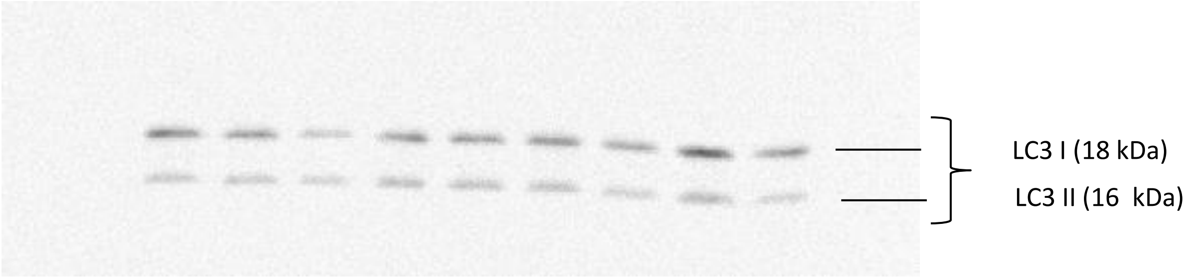

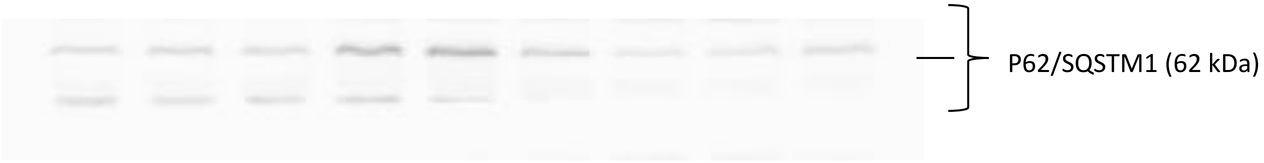

Image related to figure 10 of the manuscript (P62/SQSTM1)

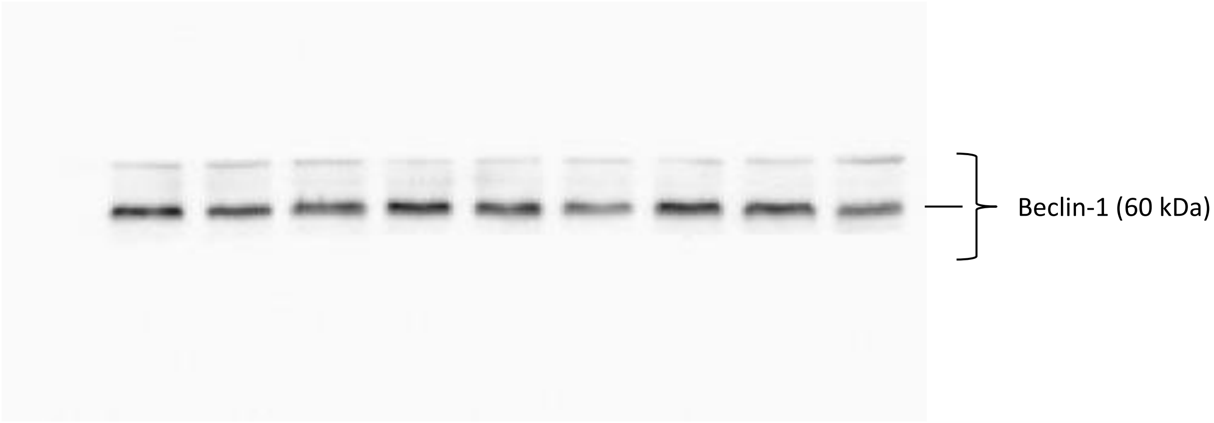

Images related to figure 10 of the manuscript (Beclin-1)

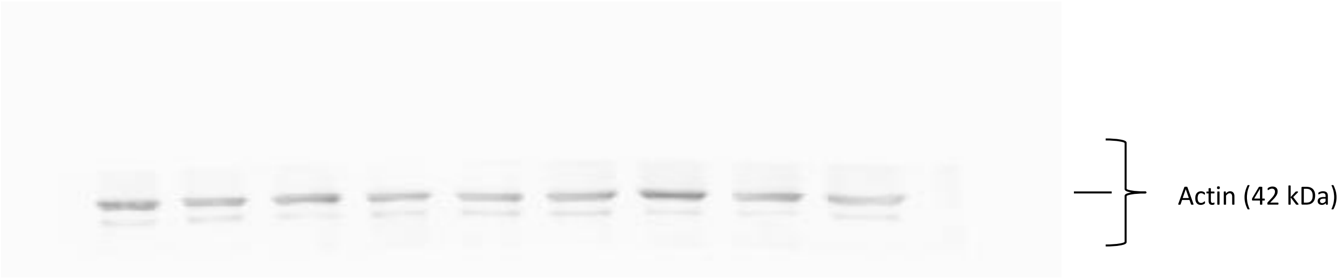

Images related to figure 10 of the manuscript (Actin)
